# Transcriptome Changes in Colorectal Cancer Cells upon Treatment with Avicequinone B

**DOI:** 10.34172/apb.2020.077

**Published:** 2020-08-09

**Authors:** Yanet Ocampo, Daneiva Caro, David Rivera, Jhoan Piermattey, Ricardo Gaitán, Luis A. Franco

**Affiliations:** ^1^Biological Evaluation of Promising Substances Group, Department of Pharmaceutical Sciences, University of Cartagena, Carrera 50 No. 29-11, 130014, Cartagena, Colombia.; ^2^Natural Products Group, Department of Pharmaceutical Sciences, University of Cartagena, Carrera 50 No. 29-11, 130014, Cartagena, Colombia.

**Keywords:** Avicequinone B, Colorectal cancer, RNA-sequencing, Interferon stimulated genes, Ferroptosis, miR-21

## Abstract

***Purpose:*** Naphtho[2,3-b]furan-4,9-dione (Avicequinone B), a natural naphthoquinone isolated from the mangrove tree *Avicennia alba* , is recognized as a valuable synthetic precursor with anti-proliferative effect. However, the molecular mechanism involved in its bioactivity has not been investigated. This study aimed to determine the selectivity of avicequinone B against cancer cells and the transcriptomic changes induced in colorectal cancer (CRC).

***Methods:*** The cytotoxic effect against adenocarcinoma-derived cells or fibroblasts was evaluated using MTT assay. In addition, CRC cells were treated with avicequinone B in different settings to evaluate colony-forming ability, cell cycle progression, apoptosis/necrosis induction, and transcriptome response by RNA-seq.

***Results:*** Avicequinone B effectively reduced the viability of breast, colorectal, and lung adenocarcinoma cells with IC_50_ lower than 10 μM, while fibroblasts were less affected. The induction of G2/M arrest and necrosis-like cell death were observed in avicequinone B-treated HT-29 cells. Furthermore, RNA-seq revealed 490 differentially expressed genes, highlighting the reduction of interferon stimulated genes and proliferative signaling pathways (JAK-STAT, MAPK, and PI3K-AKT), as well as the induction of ferroptosis and miR-21 expression.

***Conclusion:*** In short, these results demonstrated the therapeutic potential of avicequinone B and paved the foundation for elucidating its mechanisms in the context of CRC.

## Introduction

Mangrove ecosystems inhabit the intertidal regions of tropical coastlines and statuaries, typically between 30° north and south of the equator.^1^ Recently, mangroves have gain increasing attention due to their remarkable adaptability/tolerance toward hostile environmental conditions (i.e. high salinity, high temperature, muddy anaerobic soils, extreme tides, and strong winds) resulting in novel bioactive products which could be successfully used to treat human diseases, including cancer.^1,2^ However, research to exploit the potential of mangrove-derived metabolites is still limited.

*Avicennia* (Avicenniaceae) is the only mangrove genus that occurs throughout the world, comprising eight species with diverse ecological, phytochemical, and ethnomedicinal importance. Among them, *A. alba* , *A. officinalis* , and *A. marina* are considered highly valuable species due to their medicinal properties and chemical composition. In fact, several types of bioactive metabolites have been obtained from *Avicennia* , including flavonoids, tannins, terpenoids, fatty acids, and naphthoquinones.^3^ Outstandingly, this genus is documented as a rich source of naphthoquinones which are recognized by their antibacterial, antioxidant, chemopreventive, and anti-cancer activities.^4-7^ Among them, naphtho[2,3-*b* ]furan-4,9-dione (avicequinone B) and other structurally-related annulated furan derivatives such as naphtho[1,2-*b* ]furan-4,5-dione, have demonstrated outstanding cytotoxicity against cancer cells.^8-11^

Avicequinone B was first isolated by Ito *et al*^6^ in 2000 from the stem bark of *A. alba* , and later described by Jia et al^12^ in the leaves of *A. marina.* Interestingly, the bioactivity of avicequinone B was enthusiastically studied since 1994, when Koyanagi et al.^13^ proposed a reliable method for its chemical synthesis; at a time when it was consider an unnatural analogue or a mere precursor during the biosynthetic process. From then on, optimization of the preparation of avicequinone B has been a prolific field, since it is an important precursor for the synthesis of new annulated furan-naphthoquinone derivatives.^14^ In our ongoing study to optimize the structure of cytotoxic naphthoquinones against colorectal cancer (CRC), avicequinone B was identified as an effective inhibitor of HT-29 cells proliferation.^15^ In this work, we aimed to investigate the selectivity of avicequinone B towards adenocarcinoma cell lines, as well as the molecular mechanisms involved in its cytotoxicity using transcriptome sequencing. Our results provided evidence of several novel mechanisms correlated with the reduction of an inflammatory gene program, inhibition of proliferative signaling, and the induction of ferroptosis, to explain the role of avicequinone B as a promising anti-CRC drug.

## Materials and Methods

### Test compound

Avicequinone B (naphtho[2,3-*b* ]furan-4,9-dione) was synthesized as previously described by Acuña et al^15^ Analytical data confirmed that the purity of test compound was >99%. For bioassays, the compound was dissolved in dimethylsulfoxide (DMSO, Fisher Scientific, USA) and diluted in complete medium when needed.

### Cell culture

Cell lines from colorectal adenocarcinoma (HT-29), lung carcinoma (A549), prostate adenocarcinoma (PC-3), and breast adenocarcinoma (MDA-MB-231) as well as normal dermal fibroblasts (PCS-201-012), normal human fetal lung fibroblast (MRC-5), and *Mus musculus* embryo fibroblasts (3T3-L1), were obtained from the American Type Culture Collection (ATCC, Manassas, VA, USA) and cultured as detailed in supplementary methods.

### Cell viability assay

Cytotoxicity of avicequinone B was evaluated using the 3-(4,5-dimethylthiazol-2-yl)-2,5-diphenyltetrazolium bromide (MTT) reduction assay as described in supplementary methods.

### Clonogenic assay

The ability of avicequinone B to suppress the replicative capacity of HT-29 and MRC-5 cells was confirmed using the clonogenic assay.^16^ Briefly, 750 cells/well were cultured into a 6-well plate for 6 h, and treated with test compound (0-16.4 μM) or vehicle (DMSO) for 48 h. Subsequently, the culture medium was replaced and the colonies were allowed to form for 7 days. Finally, colonies were fixed, stained with 0.5% crystal violet (Sigma-Aldrich, St. Louis, MO, USA), and counted using a stereomicroscope (EZ4 HD, Leica Microsystems, Singapore).

### Cell cycle analysis

HT-29 cells (2×10^5^ cells/mL) were exposed to avicequinone B (8.20 μM) or vehicle (DMSO) for 48 h. Afterwards, the cells were harvested, washed with PBS, and fixed with cold 66% ethanol at 4°C overnight. Then, the cells were stained with propidium iodide (PI) and incubated with RNase A, according to the manufacturer’s instruction (kit ab139418; Abcam, Cambridge, UK). The effect of avicequinone B on cell cycle distribution was determined with flow cytometry (Dako, Beckman Coulter Inc., CA, USA).

### Annexin V-SYTOX flow cytometry assay 

Apoptosis/Necrosis of CRC cells was evaluated by means of the Annexin V-FITC Apoptosis Detection Kit (ab14086; Abcam, Cambridge, UK). Briefly, HT-29 cells (2×10^5^ cells/mL) were treated with avicequinone B (8.20 μM) or vehicle for 48 h. Then, the cells were harvested and stained with Annexin V-FITC and SYTOX Green dye to measure the proportion of live, apoptotic, and necrotic cells with flow cytometry ((Dako, Beckman Coulter Inc., CA, USA) ).

### Total RNA extraction and analysis

HT-29 CRC cells were treated for 48 h with avicequinone B (8.20 μM) and total RNA was extracted using the GeneJET™ RNA Purification kit (Thermo Fisher Scientific, Vilnius, Lithuania) as described by the manufacturer. The isolated RNA was analyzed at Corporación CorpoGen (Bogotá, Colombia). RNA purity and concentration were measured using a NanoDrop 2000c spectrophotometer (Thermo Scientific, Waltham, MA, USA) and a Qubit^®^2.0 Fluorometer (Life Technologies, Carlsbad, CA, USA), while RNA integrity was assessed using the Agilent 2100 Bioanalyzer system (Agilent Technologies, Santa Clara, CA, USA). Samples concentration varied between 11.4 and 97.6 ng/μL; 260/280 ratio were above 1.85; and RNA integrity number (RIN) value ranging from 4.2-9.0, with the majority of samples with RIN>7.

### RNA-sequencing analysis

Library preparation and sequencing was performed at MR DNA (www.mrdnalab.com, Shallowater, TX, USA). Total RNA (150-500 ng) was used for library construction with the TruSeq™ RNA LT Sample Preparation kit (Illumina Inc., San Diego, CA, USA), according to the manufacturer’s instructions. Subsequently, the validation of the enriched samples libraries was performed using the Qubit^®^dsDNA HS Assay Kit (Life Technologies, Carlsbad, CA, USA) and the Agilent 2100 Bioanalyzer system (Agilent Technologies, Santa Clara, CA, USA). The product was a smear ranging in size from 461-680 bp. The libraries were normalized and pooled at equimolar concentration (2 nM). Cluster generation was performed using 5 pM of pooled normalized libraries on the cBOT (Illumina Inc., San Diego, CA, USA). Finally, sequencing was performed on the Illumina HiSeq 2500 for 300 cycles (2×150 bp, paired end run) (Illumina, Inc, California, USA). The bioinformatic analysis was performed as detailed in supplementary methods.

### Quantitative real-time PCR (RT-qPCR)

To validate the RNA-sequencing analysis, six differentially expressed genes - DEGs (three up-regulated and three down-regulated) were selected for RT-qPCR, which was performed as described in the supplementary methods. Primers sequences are listed in Table S1.

### Statistical Analysis

Where appropriate, data was represented as a mean ± standard error of the mean (SEM). Data was analyzed by student *t* test or one-way analysis of variance (ANOVA) followed by Dunnett’s multiple comparisons test. Statistical significance was considered at *P* < 0.05.

## Results and Discussion

### Avicequinone B demonstrated a selective inhibitory activity against cancer cells

**Figure 1:**
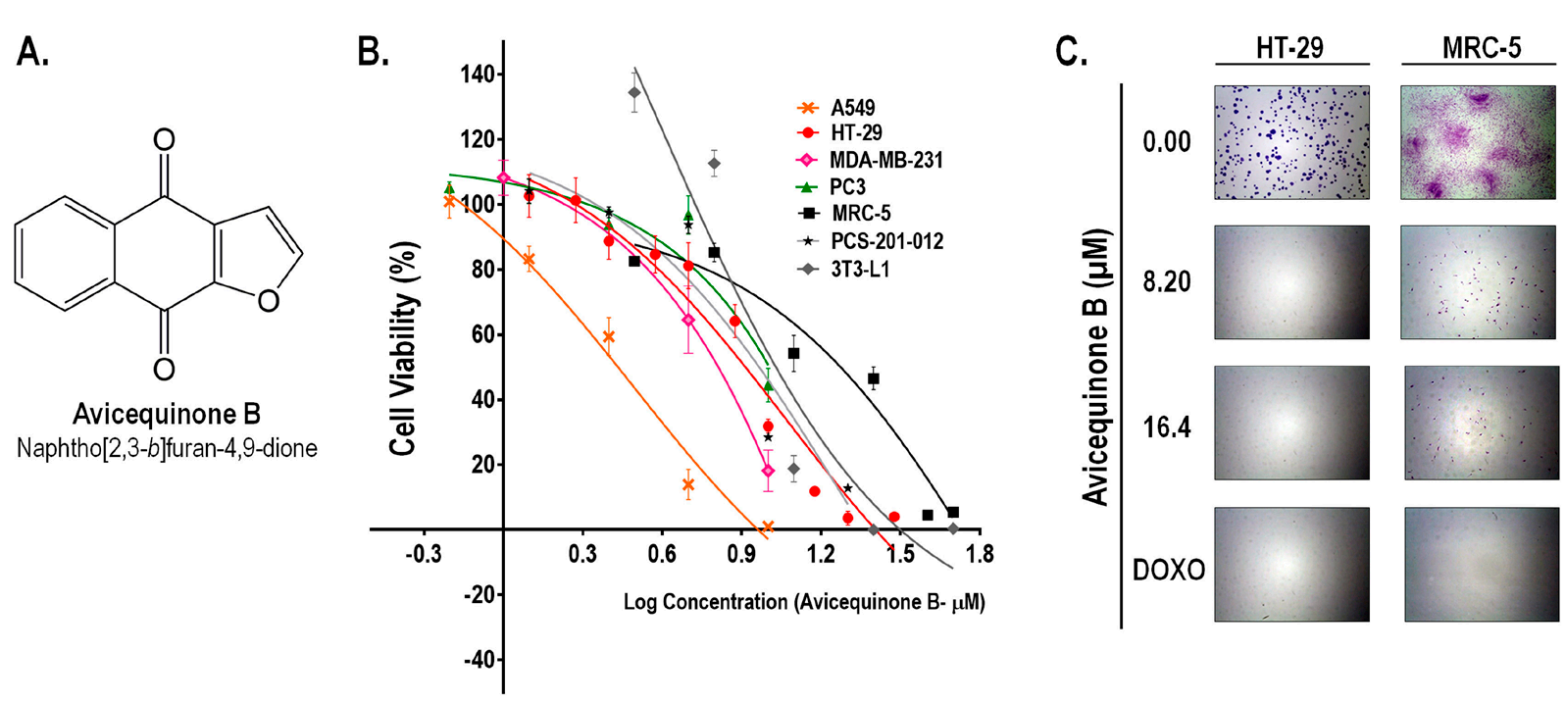
** Avicequinone B inhibits the viability and proliferation of cancer cells.** (**A**) Chemical structure of the test compound. (**B**) The cell viability of several human adenocarcinoma cells (A549, HT-29, MDA-MB-231, and PC3) was efficiently and selectively reduced in comparison to normal fibroblasts (MRC5, PCS-201-012, and 3T3-L1). The percentage of viable cells in control was set as 100 %. Results are representative of at least two independent experiments and are expressed as the mean ± S.E.M. (*n* =6-11 per data group). (**C**) The colony forming-ability of HT-29 cells was completely suppressed by avicequinone B (8.20 and 16.40 µM) whereas human normal fibroblasts were partially affected. The chemotherapeutic drug, doxorubicin (DOXO), was used as reference. Representative pictures of colonies are shown (8X).

As we recently reported,^15^ avicequinone B (naphtho[2,3-*b* ]furan-4,9-dione, Figure 1A) demonstrated a potent cytotoxic activity against CRC cells (HT-29) with an inhibitory concentration 50 (IC_50_) of 8.20±0.06 μM. Similarly, test compound affected the viability of cells from breast (MDA-MB-231; IC_50_=6.43 ± 0.28 μM), lung (A549; IC_50_=2.71 ± 0.18 μM), and prostate adenocarcinomas (PC3; IC_50_=11.65 ± 1.54 μM) (Figure 1B). The selectivity of avicequinone B was confirmed using lung fibroblasts (MRC-5; IC_50_=21.80 ± 2.68 μM), dermal fibroblasts (PCS-201-012; IC_50_=9.12 ± 0.29 μM), as well as mouse 3T3-L1 fibroblasts (IC_50_=10.71 ± 0.89 μM). Since avicequinone B and its angular analogue (naphtho[1,2-*b* ] furan-4,5-dione) have been previously identified as potent cytotoxic agents against cells from breast (i.e. MCF7 and MDA-MB-231) and lung (i.e. A549, H460, and NCI-H460) adenocarcinoma^8-11^; we continued the pharmacological study of the test compound using CRC cells.

Clonogenic assay showed that cytotoxic concentrations of avicequinone B (8.20 and 16.40 μM) suppressed the colony-forming ability of HT-29 cells, confirming its effectiveness against CRC cells (Figure 1C). In contrast, MRC-5 fibroblasts were alive and some colonies were observed; however the treatment with test compound significantly impaired their proliferative rate, raising concern about its safety. Therefore, to continue our work with avicequinone B as a potential lead compound to develop new anti-CRC drugs, a battery of three *in vitro* genotoxicity tests (Ames test, Micronucleus assay, and Comet test) was carried out as described in supplementary methods. Results evidenced the lack of mutagenicity of avicequinone B when employing its effective cytotoxic concentration (IC_50 _= 8.20 μM) (Figure s1).

### Anti-proliferative effect of avicequinone B occurs by arresting cell cycle progression and inducing cellular death

**Figure 2:**
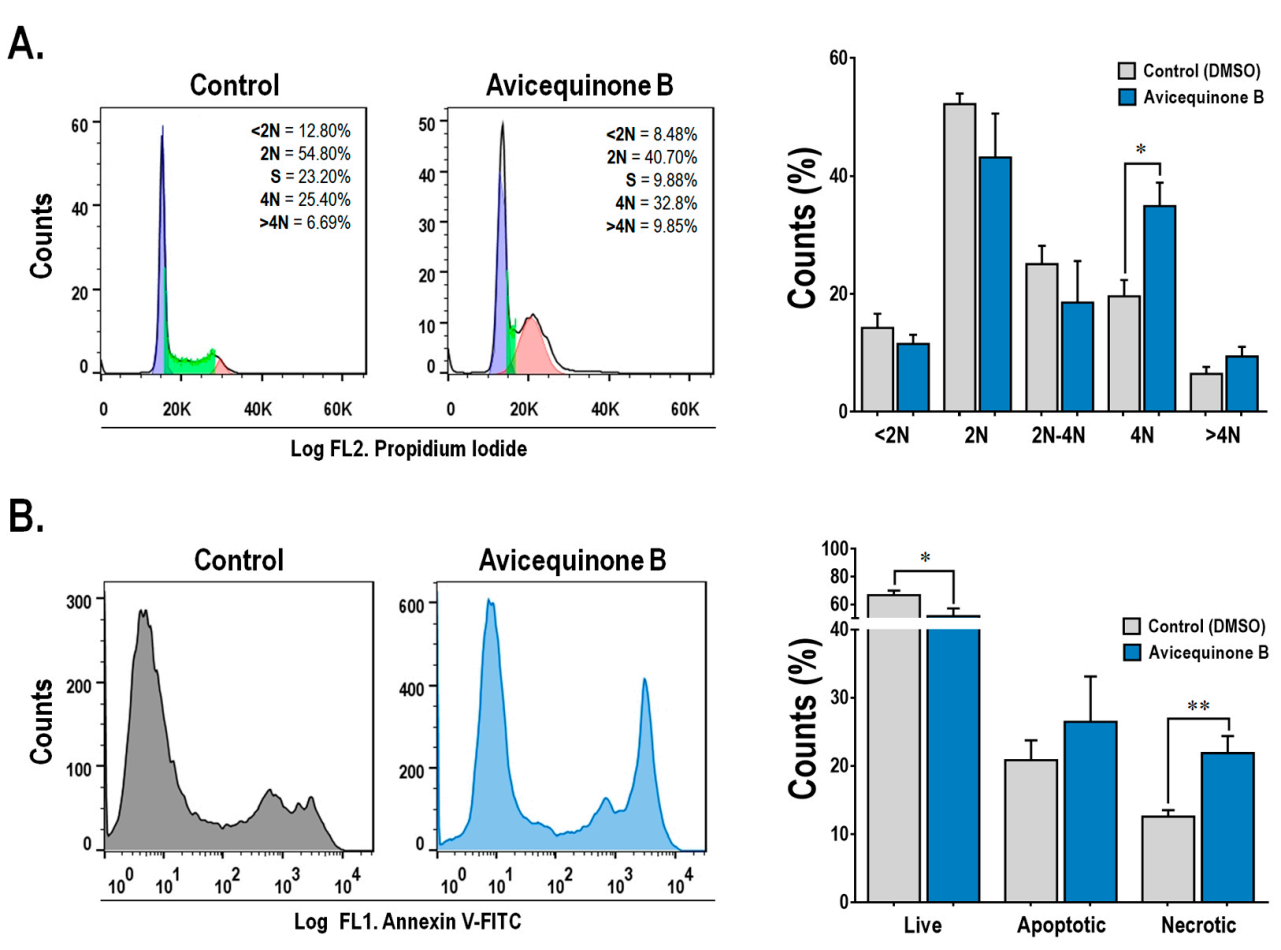
** Avicequinone B induces cell cycle arrest and necrosis-like cell death.** Flow cytometry data showed that (**A**) HT-29 cells were accumulating at the G2/M phase (4N) and (**B**) undergoing cell death through necrosis, when treated with avicequinone B (8.20 µM) for 48 h. Distinctive histograms are presented showing the number of cells (vertical axis) vs. DNA content-propidium iodide or Annexin-V/Sytox green staining (horizontal axis-FL1 and FL2 channel, respectively). Results are representative of at least three independent experiments and are expressed as the mean ± S.E.M. (n=3-10 per group). Significant differences from the control are indicated by *P <0.05; **P <0.01 (ANOVA).

As can be seen in Figure 2A, the treatment with avicequinone B, resulted in a significant increase in the proportion of 4N cells (G2/M phase) from 19.58±2.79% (control-0 μM) to 34.90±3.98% (avicequinone B-8.20 μM), whereas 2N (G1) and 2N-4N (S) cell populations tended to be reduced. Thus, avicequinone B triggers the arrest at the G2/M phase in HT-29 cells. Consistently, Tseng et al^9^ reported G2/M arrest when K562 leukemia cells were treated with this compound.

On the other hand (Figure 2B), the staining with Annexin V-FITC and SYTOX demonstrated that avicequinone B (8.20 μM) enhanced the proportion of highly intense green fluorescence HT-29 cells (indicative of necrosis), without significantly increasing apoptotic cells (positive green fluorescence). This result differs from that obtained by Prateep et al^8^ who reported the induction of apoptosis, but not necrosis, in H460 cells treated with avicequinone B. This discrepancy might be explained by the differences in the experimental setting, since they used a mild test concentration (lower than the IC_50_).

### Transcriptome sequencing revealed that avicequinone B promoted cellular death mainly by reducing the expression of IFN-stimulated genes.

**Figure 3:**
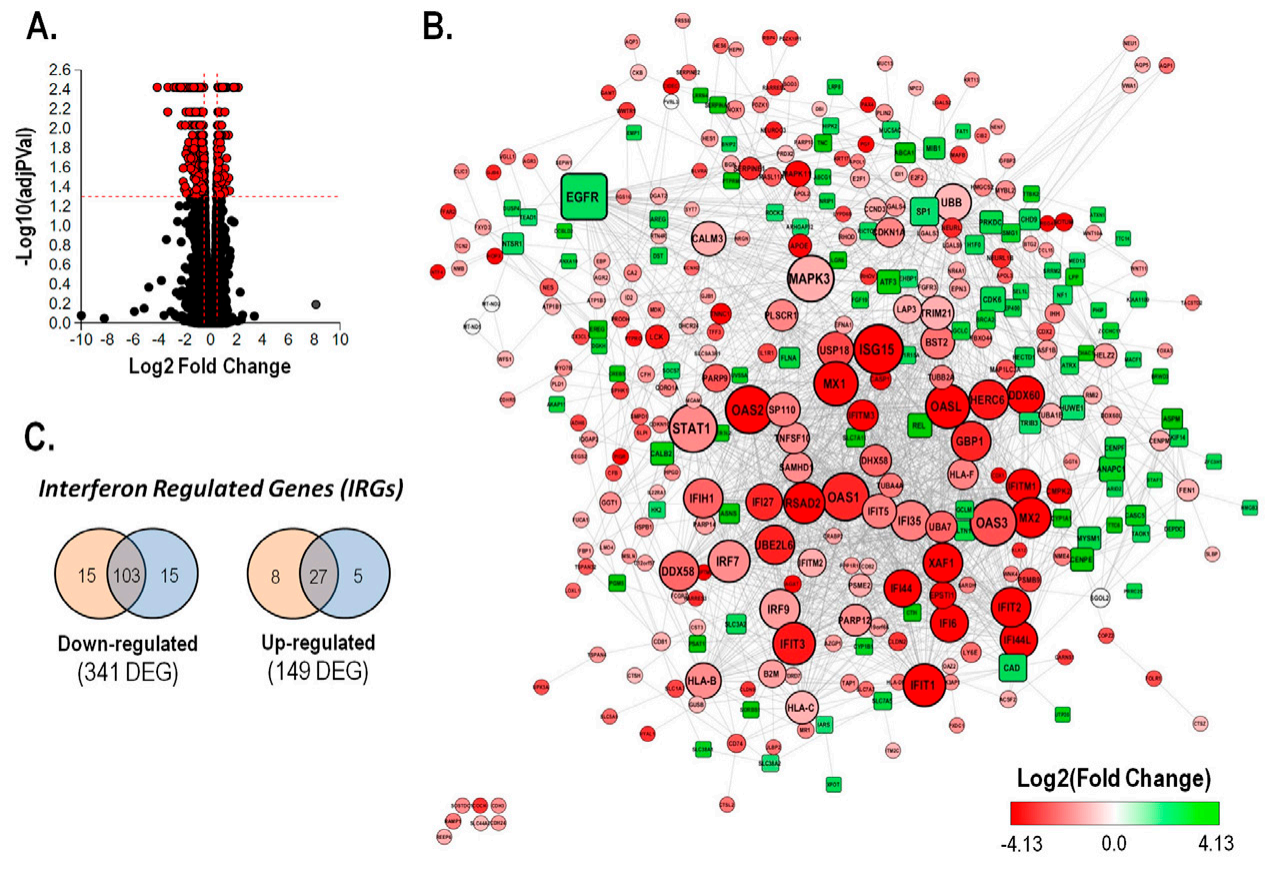
** Treatment with avicequinone B reduced the inflammatory transcriptome signature of HT-29 CRC cells by reducing the expression of interferon regulated genes.**Transcriptomic analysis was performed by means of RNA-sequencing (**A**) Volcano plot presenting the mRNA expression changes of HT-29 cells treated with avicequinone B (8.20 μM) compared to control. All measured genes (dots) are represented in terms of their calculated expression change (x-axis) and the significance of the change (y-axis). The significance is represented in terms of the negative log (base 10) of the *p* -value, so that more significant genes are plotted higher on the y-axis. The dotted lines represent the thresholds used to select the differentially expressed genes (DEGs): 0.5 for expression change and 0.05 for significance. DEGs are presented as red dots (490). (**B**) The protein-protein interaction network of DEGs was built using STRING database at medium confidence (score=0.4) and analyzed using Cytoscape. Each node represents a gene/protein, the edge represents the interaction between gene/proteins, the size of the node is proportional to the degree of the node, the shape indicates the direction of the expression (square: up-regulation and circle: down-regulation), and the color represents the Log2 Fold Change. (**C**) Number of type I (orange) and type II (blue) interferon regulated genes (IRGs) that were identified in the list of DEGs regulated by avicequinone B using the Interferome database, filtering species (*Homo sapiens* ) and system (gastrointestinal tract).

To evaluate the mechanism involved in the bioactivity of avicequinone B against CRC, an RNA sequencing (RNA-seq)-based transcriptomic analysis was carried out using HT-29 cells treated with vehicle (control) or a toxic concentration of test compound (8.20 μM). The summary of the RNA-seq data showed similarity of reads, mapping rates, and GC content within the independent analyzed samples (Table S2, n = 3). In this experiment, 490 differentially expressed genes were identified out of a total of 12761 genes with measured expression (Figure 3A). These were obtained using a threshold of 0.05 for statistical significance (*p-* and FDR -*q* - values) and a log fold change of expression with absolute value of at least 0.5. Avicequinone B significantly down-regulated the expression of 341 genes while 149 genes were up-regulated (Table S3). The expression levels of a subset of DEGs, 3 up-regulated (SPRR1B, AKAP12, CYP1A1) and 3 down-regulated (IFITM1, IFI44L, EPSTI1), were measured via RT-qPCR to confirm the reliability of the transcriptomic analysis. The RT-qPCR results were consistent with those of the RNA-seq, showing a significant correlation (least-squares linear regression, *P* < 0.05; R^2^= 0.909-0.935; Figure S2), thus validating the analysis.

The expression level of DEGs was integrated within a protein-protein interaction network built with STRING database (interaction score >0.4). Hub genes in the network were identified by topological analysis using the Cytoscape Network Analyzer. As shown in Figure 3B, most of the nodes with higher degree (ISG15, STAT1, OAS1, OAS2, OAS3, MAPK3, EGFR, MX1, OASL, and IFIT1) are interferon regulated genes (IRGs) whose expression was reduced by avicequinone B. Among these, STAT1, ISG15, MAPK3, and to a lesser extent OAS2 presented the highest centrality scores. Moreover, nearly 39% of the down-regulated DEGs are predicted to be regulated by type I and/or type II interferon (Figure 3C), as classified in the Interferome database. Likewise, out of the top 10 down-regulated genes (Table S4), seven were identified as IRGs (IFITM1, IFI44L, RSAD2, EPSTI1, TNNC1, GPRC5B, and CMPK2).

Simultaneously, the analysis of the top 10 up-regulated genes by avicequinone B (Table S5) showed that some were associated with the cornified envelope, epidermis development and cellular junction (SPRR1B, SCEL, and TNC); detoxification (CYP1A1), and tumor suppression in CRC (AKAP12). In addition, avicequinone B induced the expression of genes with low abundance mRNA in affected tissue or cells isolated from CRC patients (SLC7A11, SCEL, LRRN4, and SERPINA3). From this analysis, it was also noted that avicequinone B increased the expression of two markers of CRC metastasis: HOPX (down-regulated) and TNC (up-regulated), probably as a survival response of injured HT-29 cells.

**Figure 4:**
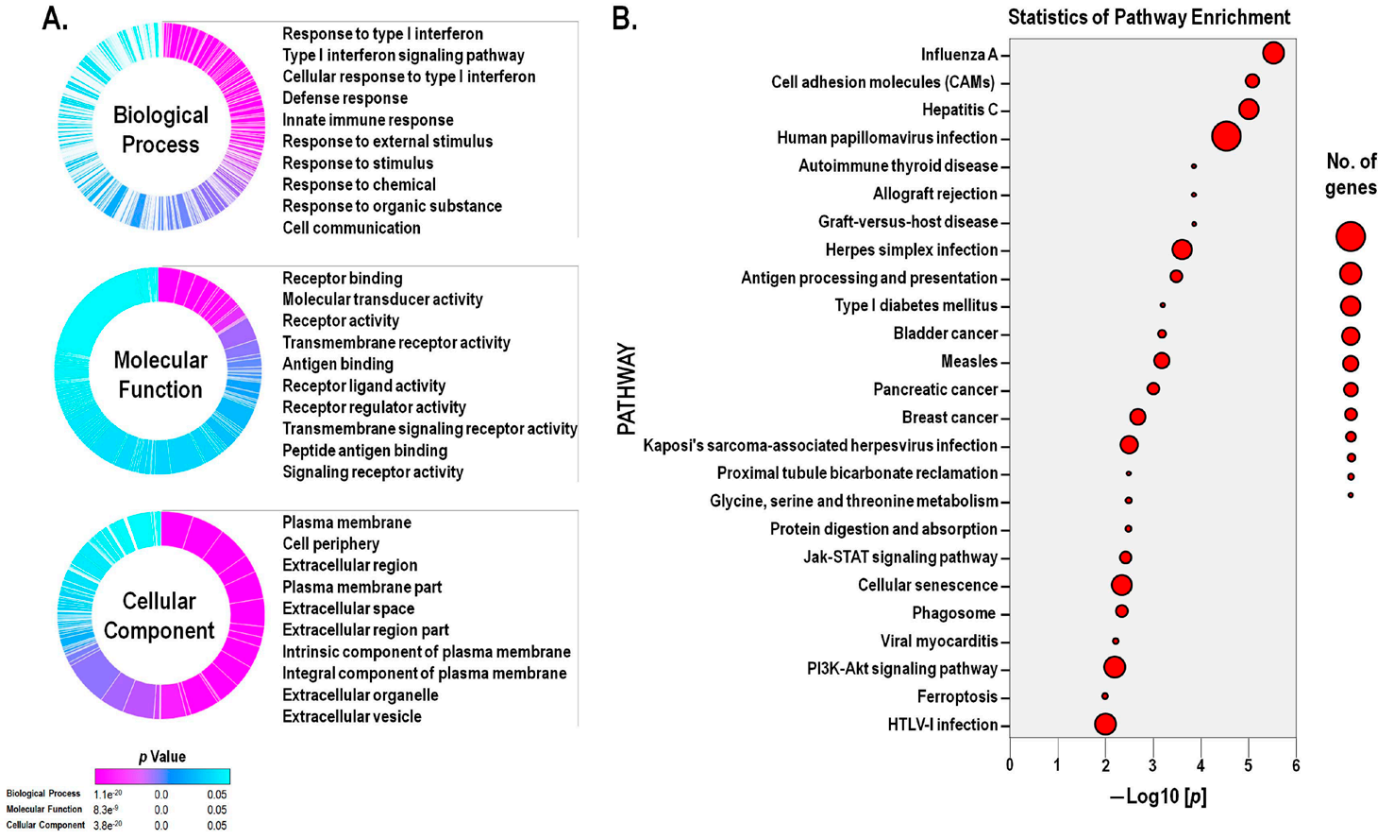
** Avicequinone B promoted alterations in the gene expression associated with cellular survival, proliferation, junction and signaling; response to interferon and virus; cancer pathways; and ferroptosis.** (**A**) Differentially expressed genes (DEGs), between avicequinone B (8.20 µM)-treated HT-29 cells and control (vehicle), were categorized within three domains: biological processes, molecular function, and cellular components, through gene ontology (GO) term enrichment analysis performed with iPathwayGuide online software; cut-off *p* value *p* <0.05 corrected for multiple comparisons using FDR and Bonferroni. The GO summary graphs plot the significant overrepresented terms for each domain. Each section of the graph represents a different term, the height of the section is proportional to the number of DEGs, and the enrichment significance is displayed using a color scale with cyan being least significant and magenta most significant. (**B**) Scatter plot of the top twenty-five significantly enriched pathways for DEGs, as categorized by iPathwayGuide online software. Pathways are ranked (x-axis) according to their significance (y-axis) which is represented in terms of the negative log (base 10) of the *P* value (without correction). The size of the dots represents the number of DEGs.

Gene expression data was examined in the context of pathways, gene ontologies, and miRNAs, using the iPathway online software. As a result, 49 pathways were found to be significantly impacted. In addition, 1684 gene ontology (GO) terms and 5 microRNAs (miRNAs) were found to be significantly enriched based on uncorrected *p* -values. Figure 4A summarizes the significantly enriched GO terms related to biological process, molecular function, and cellular component, emphasizing the top-ten terms. Results demonstrated that avicequinone B induced a significant enrichment of terms related to the onset or progression of cancer such as cellular proliferation, cellular differentiation, cellular migration, angiogenesis, regulation of programmed cell death, and apoptotic process. Moreover, terms related to cell-cell signaling, cell-cell adhesion, assembly of the plasma membrane and extracellular space, response to drugs, and response to oxidative stress were also enriched. In particular, the treatment with avicequinone B promoted the differential expression of genes associated to immune response terms related to interferon (IFN) signaling, response to IFNs, and response to virus. Furthermore, the impact analysis revealed that among the top twenty-five influenced pathways (Figure 4B); eight are related to viral infections (influenza A, hepatitis C, human papillomavirus infection, herpes simplex infection, measles, Kaposi’s sarcoma-associated herpes virus infection, viral myocarditis, and HTLV-I infection). This distinctive association of avicequinone B with terms and pathways related to immune response to virus is explained by the remarkable down-regulation of IRGs, as described above.

**Figure 5:**
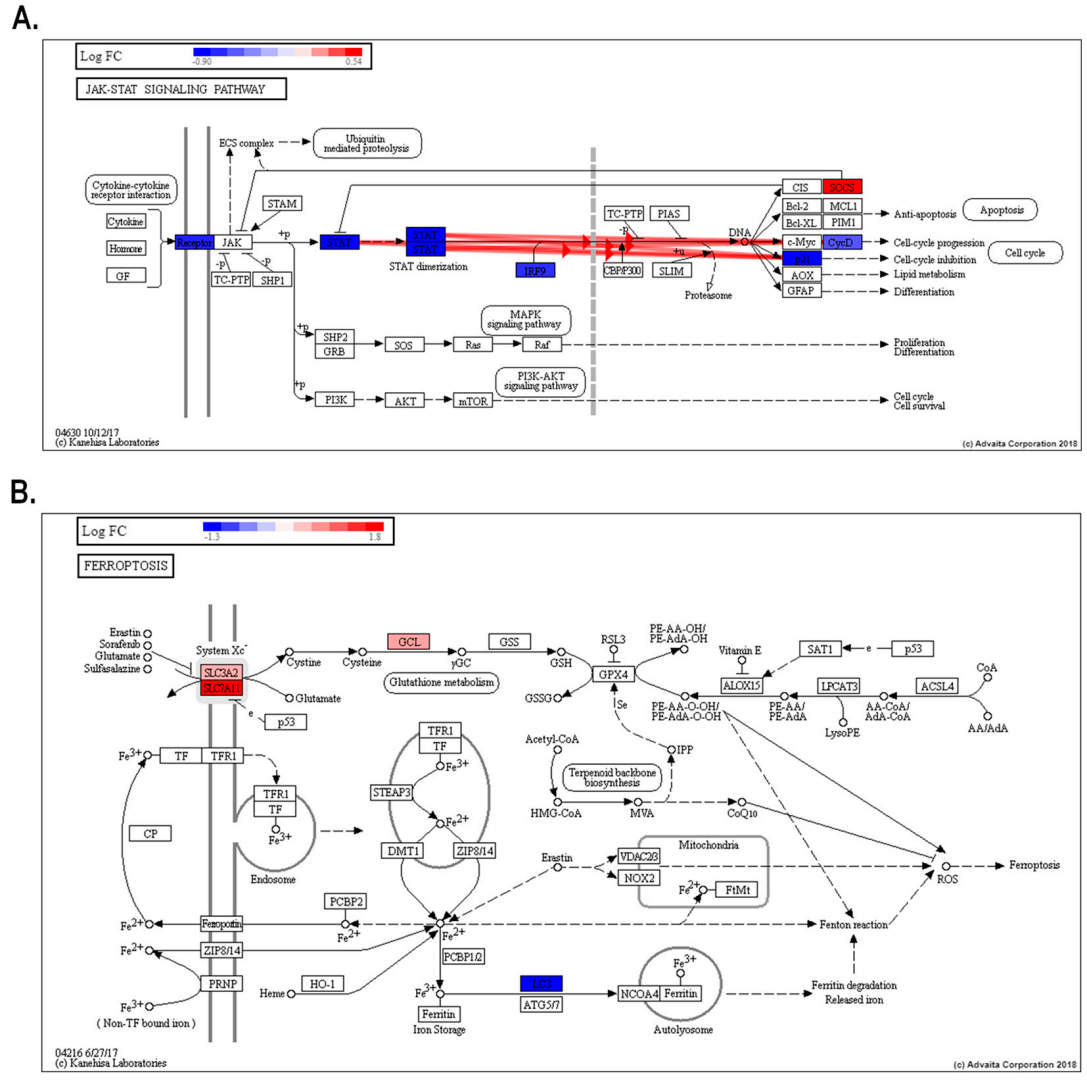
** JAK-STAT and ferroptosis signaling pathways are important for avicequinone B-induced cytotoxicity of CRC cells.** Treatment with avicequinone B (8.20 µM) significantly impacts the expression of genes involved in JAK-STAT and ferroptosis signaling pathways, both included in the top twenty-five impacted pathways. (**A**) The inhibition of JAK-STAT pathway might explain the remarkable suppression of interferon regulated genes (IRGs); (**B**) while cellular death trough ferroptosis fits with the increased Sytox staining and oxidative stress induced by test compound. Impact analysis was performed using iPathwayGuide online software. The heat map at the top left shows the Log2 fold change (LogFC), where blue indicates down-regulated genes and red indicates up-regulated genes. Only those genes that meet our criteria of differential expression are shown (Log2ǀ(fold change)ǀ≥0.5, *P* value ≤ 0.001, and *q* -value ≤ 0.05). The bold red highlighted arrows indicate coherent cascades.

Additionally, avicequinone B significantly impacted five cancer pathways -including bladder cancer (KEGG: 05219), pancreatic cancer (KEGG: 05212), and breast cancer (KEGG: 05224) (Figure S4)- displaying a shared differential expression of genes related to cellular proliferation, differentiation and/or migration (ERK and EGFR), as well as cell cycle progression (p21 and E2F). Interestingly, the test compound down-regulated oncogenes (FGFR3 and HES1) and genes belonging to the WNT family, while up-regulated tumor suppressor genes (BRCA2). Gene pathway analysis also revealed that avicequinone B impacted pathways related to development and homeostasis (JAK-STAT signaling pathway-KEGG: 04630) (Figure 5A); regulated cell death (ferroptosis- KEGG: 04216) (Figure 5B); cellular interactions (cellular adhesion molecules-KEGG: 04514); cellular proliferation and survival (PI3K-AKT signaling pathway-KEGG: 04151; and MAPK signaling pathway-KEGG: 04010) (Figure S5 and S6).

Collectively, these results suggest that IRGs might play a central regulatory role in the bioactivity of avicequinone B. Over the past decades, the idealization of IFNs, especially INF-γ, as an immunologic guardian against malignant neoplastic disease has changed.^17^ As a fact, IFNs act directly on cancer cells to inhibit proliferation, modulate apoptosis, improve antigen expression, and up-regulate immune interacting molecules; which is complemented indirectly by activating immune cells, inhibiting immune-suppressive cells, and promoting the switch of tumor associated macrophages (TAMs).^18^ However, like most cytokines, IFNs induce feedback inhibitory mechanisms to restrain the magnitude of immune responses to avoid the destruction of tissues; therefore, in the scenario where the suppression overcomes the activating mechanisms, IFNs might enhance proliferative and anti-apoptotic signals in tumor cells providing a survival advantage to cancer cells. Indeed, in tumors IFN-γ induces the expression of inhibitory receptors, such as programmed cell death 1 ligand 1 (PDL1) on tumor cells and TAMs or suppressor of cytokine signaling 2 (SOCS2) in dendritic cells.^19^ In agreement, several recent studies demonstrate the association of treatment-resistant cancer cells and the overexpression of IRGs.

The striking emergence of IFNs and IRGs as important players in the progression and the resistance of cancer cells have gained interest, especially in the context of CRC, since increasing evidence reveals that affected tissue or cells from patients exhibit overexpression of IRGs when compared to normal samples. For example, IRGs such as CMPK2,^20,21^ IFITM1,^22-26^ IFITM2,^22,27^ IFITM3,^22,28^ IRF2,^29^ ISG15,^30^ MX1,^31,32^ and PLSCR1,^33^ have been proposed as markers for diagnosis, aggressiveness and prognosis of CRC. Interestingly, the treatment of HT-29 cells with avicequinone B diminished the expression of several CRC-related IRGs (CMPK2, IFIT1, IFITM1, IFITM2, IFITM3, ISG15, and PLSCR1) together with other cancer-related IRGs (IFI27, IFIT2, IFIT3, IRF7, IRF9, and STAT1).

Although in several cases the cellular source of IFNs or IRGs has not been well-defined, intestinal epithelial cells play an important role as producers of inflammatory cytokines (including IFNs) to stimulate anti-viral response among other cellular function,^34^ and might be speculated that its sustained production could initiate and maintain tumor growth. In agreement, Wagner et al^32^ recently identified that the elevated expression of inflammatory/IFN-regulated genes (i.e. IFIT1, MX1, IFI44L, among others) is the key factor associated with the intrinsic or acquired resistance of KRAS-mutant epithelial CRC cells to mitogen-activated protein kinase (MEK) inhibitors (MEKi), thus explaining the clinical failure of the MEKi trametinib to treat KRAS or BRAF-CRC, while thriving as BRAF-mutant melanoma therapy. Moreover, their experimental data confirmed that the intrinsic inflammatory environment in the colon, which is intensified by oncogenic KRAS and chemotherapy, results in a tumor microenvironment driven by a persistent inflammatory/IFN transcription program that operates to provide resistance to CRC cells allowing them to survive and proliferate. Hence, the successful treatment of CRC, both *in vitro* and *in vivo* , with anti-proliferative compounds (bromodomain inhibitors) involved the suppression of IRGs.

Similarly, the effective cytotoxicity against HT-29 cells exerted by avicequinone B, involved a broad inhibition of IRGs expression, which was also accompanied by a significant impact to the canonical Janus kinase (JAK)–signal transducer and activator of transcription (STAT) signaling pathway, known to be activated by IFNs and other cytokines (Figure 5A). In normal cells, the activation of JAK-STAT signaling is short and quick, whereas its constitutive activation/overexpression leads to oncogenic transformation, tumor cell invasion, and metastasis.^35^ In particular, elevated expression/activation of JAK, STAT, IRF9, as well as the inhibition of suppressor of cytokine signaling proteins (SOCS), is reported for various cancer types, including CRC.^36-39^ In HT-29 cells, the treatment with avicequinone B significantly reduced the expression of various proteins involved in this signaling pathway, from receptor to target genes, while inducing the expression of SOCS. Remarkably, naturally occurring naphthoquinones, structurally related to avicequinone B (i.e. substituted furan-fused naphthoquinones), have been recently reported as STAT3 inhibitors with strong anti-proliferative activity,^40^ thus supporting our findings. Nonetheless, whether avicequinone B inhibits STAT directly or through a regulator remains to be determined. Overall, this evidence indicates that avicequinone B significantly inhibits the core JAK-STAT-IRGs response, resulting in a distinctive down-regulation of IRGs as an important mechanism for it anti-proliferative effect against HT-29 cells.

In addition, avicequinone B significantly impacted other signaling pathways related to cellular proliferation and survival such as phosphatidylinositol-3’-kinase (PI3K)/serine-threonine kinase (AKT) pathway and the mitogen-activated protein kinase (MAPK) pathway. In this regard, the extracellular signal-regulated kinase (ERK) was identified as an important molecular target suppressed by avicequinone B. Notably, ERK activation/expression is involved in the cellular response to environment regulating cell cycle and cellular differentiation and senescence, which is critical for cancer initiation and progression (Figure S4). In agreement, Patreep et al^8^ demonstrated that the inhibition of ERK phosphorylation by avicequinone B is an important mechanism involved in the induction of detachment-induced cell death, also known as anoikis, in resistant lung cancer cells. Taken together, this experimental evidence demonstrates that ERK-related signaling pathways play an important role in the cytotoxic effect of the test compound against cancer cells.

Another pathway impacted by avicequinone B was ferroptosis, an iron-dependent non-apoptotic and non-necrotic oxidative form of programmed cell death that involves lethal, iron-catalyzed lipid peroxidation. Ferroptosis can be induced by two classes of small-molecule substances known as class 1, inhibitors of the cystine/glutamate antiporter (system X_c_^−^ SLC3A2/SLC7A11) that reduces glutathione (GSH) content, and class 2 or inhibitors of the glutathione peroxidase 4 (GPx4).^41^ From our results (Figure 5B), it appears that oxidative cell death induced by avicequinone B might be a consequence of (1) a decrease in the X_c_^−^ system function (decrease GSH and induces SLC7A11/SLC3A2) or (2) the induction of oxidative stress, through redox cycling, with the consequent GSH depletion and glutamate cysteine ligase (GLC) overexpression; which are both accompanied by (3) increase of intracellular iron as suggested by the down-regulation of LC3A and the up-regulation of ferritin pseudogenes (FTH1P10 and FTH1P20). To note, avicequinone B shifted the expression of SLC7A11, SLC3A and LC3 in the opposite direction to the alterations found in CRC.^42-44^ Furthermore, avicequinone B also reduced the expression of HSPB1 (also known as HSP-27), a heat shock protein associated to chemotherapy resistance and poor prognosis in CRC.^45,46^ Interestingly, HSPB1 protects cells from oxidative stress by reducing cellular iron uptake^47,48^; likewise its suppression sensitizes cancer cells (HeLa, U2OS, and LNCaP) and human xenografts mouse model to erastin-induced ferroptosis.^49^ In addition to our transcriptomic analysis, the increased influx of SYTOX Green, a membrane-impermeable nucleic acid dye that labels cells with ruptured plasma membranes, is consistent with the induction of ferroptosis, as previously described by Kim et al. ^50^ Collectively, our results suggest that avicequinone B might induce ferroptosis as a novel mechanism to inhibit cellular proliferation.

Alternatively, the analysis of the gene expression data predicted the presence of 5 miRNAs (hsa-miR-590-5p; hsa-miR-21-5p; hsa-miR-329-3p; hsa-miR-362-3p; and hsa-miR-1306-5p) based on enrichment of differentially down-regulated target genes. Among them, hsa-miR-21-5p arises as a noteworthy molecular target for avicequinone B. Although the prediction of this miRNA might appear as a deleterious side-effect of avicequinone B treatment, since it is a well-characterized oncogenic miRNA in CRC^51^; it could also indicate that test compound is inducing a severe cellular stress that might stimulate its production as a compensatory mechanism^52^; or that avicequinone B might be promoting a direct induction of miR-21-5p sensitizing HT-29 cells to death. This is supported by experimental evidence of CRC cells that exhibited higher sensitivity to chemoradiation treatment by overexpression of miR-21-5p.^53^ Furthermore, berberine (a natural isoquinoline alkaloid) suppressed growth and induced apoptosis in HepG2 cells, through miR-21 overexpression.^54^

## Conclusion

To sum up, our data demonstrated the cytotoxicity of avicequinone B, a natural furan-fused naphthoquinone from *Avicennia alba,* and its selectivity against several adenocarcinoma cell lines, including HT-29 CRC cells. In this cell line, the anti-proliferative effect of avicequinone B was induced by cell cycle arrest at G2/M and necrosis-like cell death by the induction of an anti-inflammatory gene expression program with special suppression of IRGs probably through blockade of the JAK-STAT-IRGs pathway, combined with the inhibition of proliferative signaling, as well as the induction of ferroptosis and miR-21 expression. Taken together, these results provide novel mechanisms involved in the potential anti-cancer effect of avicequinone B, as well as other substituted fused-naphthoquinones, encouraging further studies to validate its activity using *in vivo* models.

## Ethical Issues

Not applicable.

## Conflict of Interest

The authors declare no conflict of interest.

## Acknowledgments

This research was funded by Colciencias and the University of Cartagena (Grant 110756933930-2012 to LF and RG). The authors thank the Institute for Immunological Research of the University of Cartagena for their generous assistance with flow cytometry, as well as Carlos Rojas, Julián Nova, Mauro Narváez, and Jesús Cantillo for their help with genotoxicity evaluation.

## Supplementary Materials


Supplementary file 1 contains supplementary methods, Tables S1-S5 and Figures S1-S6.
