# Supplementary material for: Transcriptome Changes in Colorectal Cancer Cells upon Treatment with Avicequinone B
Source: Adv Pharm Bull. 2020 Aug 9;10(4):638–47. doi: 10.34172/apb.2020.077 (PMC7539315; doi:10.34172/apb.2020.077)
Supplement: Supplementary file 1 — contains supplementary methods, Tables S1-S5 and Figures S1-S6. [file apb-10-638-s001.pdf]

Supplementary File 1

Supplementary Methods

Cell culture

Cells were obtained from the American Type Culture Collection (ATCC, Manassas, VA, USA) and maintained with 5% CO_2 at 37 °C in McCoy's 5A medium (HT-29), Dulbecco's Modified Eagle's medium (A549, PC-3, MDA-MB-231, PCS-201-012 and 3T3-L1) or Eagle's Minimal Essential Medium (MRC-5) (Sigma-Aldrich, St. Louis, MO, USA) supplemented with 10% fetal bovine serum (FBS, Gibco, Sao Paulo, Brazil) and 1.5 g/L sodium bicarbonate (Sigma-Aldrich, St. Louis, MO, USA), plus low serum growth supplement (Invitrogen, Waltham, MA, USA) for PCS-201-012.

Cell Viability assay

Briefly, cells were seeded at appropriate densities (10.000-15.000 cells/well) in 96-well plates, incubated for 24 h, and treated with various concentrations of avicequinone B (0-50 μM). After 48 h, the cells were incubated with 0.25 mg/mL MTT (Calbiochem, La Jolla, CA, USA). Four hours later, formazan crystals were dissolved in DMSO and the O.D. at 550 nm was measured using a plate reader (Multiskan GO, Thermo Scientific, Rockford, IL, USA). Inhibition of cell proliferation was expressed as percentage of viable cells in treated samples as compared to vehicle-treated cells, and the 50% inhibitory concentration of cell proliferation (IC_{50}) was calculated by nonlinear regression.

Bioinformatics

Initial bioinformatics analysis was performed at Corporación CorpoGen (Bogotá, Colombia). First, sequencing quality was verified with the raw FastQC reads. Data pre-

Supplemental Data

processing included removing adapter sequences, trimming low quality ends, and filtering low quality reads at phred quality score Q33 using Trimmomatic.[1] Clean high-quality reads were aligned to a human reference genome (GRCh38.p7) with Tophat.[2] The expression level for each transcript was calculated as fragments per kilobase of transcript per million mapped fragments (FPKM) using Cufflinks. Differentially expressed genes (DEGs) were detected with Cuffdiff.[3] Genes were considered differentially expressed using the following threshold: log2 |(fold change)| ≥0.5, p-value ≤ 0.001, and false discovery rate (FDR) q-value ≤ 0.05. Finally, the data (significantly impacted pathways, biological processes, molecular function, cellular components, etc.) were analyzed using iPathwayGuide online software (Advaita Corporation, Plymouth, MI, USA). The protein-protein interaction network was analyzed using STRING database[4] and visualized using Cytoscape.[5]

Quantitative real-time PCR (RT-qPCR)

To validate the RNA-sequencing analysis, six DEGs (three up-regulated and three down-regulated) were selected for RT-qPCR. For each sample, 1.0 μg of RNA was employed for cDNA synthesis using the High Capacity cDNA reverse transcription kit (Applied Biosystems, Vilnius, Lithuania). RT-qPCR analysis was performed with the LightCycler® 96 System (Roche, Mannheim, Germany) using FastStart Essential DNA Green Master (Roche, Mannheim, Germany) and specific primers (**Supplementary Table 1**, Eurofins Genomics, Huntsville, USA). Gene expression was normalized to glyceraldehyde-3-phosphate dehydrogenase (GAPDH) or hypoxanthine phosphoribosyltransferase 1 (HPRT1) as endogenous housekeeping genes. Duplicate cycle threshold (CT) values were analyzed by the comparative CT (ΔΔCT) method.

Ames Mutagenicity Test

Supplemental Data

Mutagenicity of avicequinone B was assessed by using the commercial Muta-ChromoPlate™ Ames test kit (EBPI Inc., Ontario, Canada), which follows Ames´s test principle and uses *Salmonella typhimurium* (TA100). Avicequinone B was dissolved in DMSO, diluted in growth media to obtain the desired concentration (8.20 μM), mixed with *S. typhimurium* inoculum, and incubated in both presence and absence of S9 fraction, for 3 days at 37 °C in sterile air-tight plastic bag. After incubation, microplates were scored visually: yellow, partially yellow or turbid wells were scored as positives and all purple wells were scored negative. Sodium azide and 2-aminoanthracene were employed as positive control and DMSO was employed as negative control.

Micronucleus assay

3T3-L1 fibroblasts (75.000 cells/well) were seeded and incubated for 24 h, and treated with vehicle (control), avicequinone B (10.7 μM) or mitomycin C (MMC, 0.5 μM) for 30 h. Subsequently, cells were treated with cytochalasin B (4.5 μg/mL) for 18 h and then detached with trypsin, washed and re-suspended with PBS, and finally fixed with ethanol:acetic acid (3:1). Smears were prepared for each sample (20 μL), air dried, stained with Wright´s solution (Merck, Darmstadt, Germany), and labeled with a random number to avoid the identification of the treatment group during analysis. Coded slides were analyzed by two independent investigators using a Zeiss Axio Lab.A1 microscope (Carl Zeiss, Oberkochen, Germany).

Comet Assay

The comet assay was performed in neutral conditions as described by Trevigen (Trevigen, Gaithersburg, USA) with some modifications. Briefly, 3T3-L1 cells (250.000 cells/well) were treated with vehicle (control) or avicequinone B (10.7 μM) for 48 h. Hydrogen peroxide (150 μM) was employed as positive control. Cells were detached using trypsin, centrifuged, and suspended in cold PBS. The cellular suspension

Supplemental Data

(10 μL) was mixed with low-melting point agarose (Trevigen) and spread on CometSlides™ (Trevigen). After lysis, slides were equilibrated and electrophoresis was performed at 300 mA for 1 h. Finally, DNA was precipitated, and the slides were air dried, fixed in ethanol, and stained with SYBR® Safe DNA stain (Invitrogen, Carlsbad, USA). Cells were then visualized using a fluorescent microscope (Axio Lab.A1, Carl Zeiss, Oberkochen, Germany). Representative pictures were recorded at 10X magnification with a Zeiss AxioCAM digital camera (ICc5).

To assess the extent and distribution of DNA damage a qualitative analysis was performed by three blinded scorers that examined 100-1000 randomly selected and non-overlapping comets per well that were classified visually according to tail size ranging from undamaged (class 0) to maximum DNA damage (class 4), as shown in **Figure S1C**.

Supplemental Data

Supplementary Tables

Table S1. Sequences of Primers used for Real-time qPCR analysis.

Gene Symbol	Gene ID	Official Name	Primer sequences [a]	Tm [b]	Amplicon Size (pb)
AKAP12	9590	A-kinase anchoring protein 12	F: GAGATGGCTACTAAGTCAGCGG<br>R: CAGTGGGTTGTGTTAGCTCTTC	60.3 ºC<br>59.2 ºC	124
CYP1A1	1543	Cytochrome P450 family 1 subfamily A member 1	F: TCCGGGACATCACAGACAGC<br>R: ACCCTGGGGTTCATCACCAA	61.9 °C<br>61.1 °C	184
EPSTI1	94240	Epithelial stromal interaction 1	F: ACCCGCAATAGAGTGGTGAAC<br>R: GCTATCAAGGTGTATGCACTTGT	60.3 °C<br>59.1 °C	206
GAPDH	2597	Glyceraldehyde-3-phosphate dehydrogenase	F: TCGACAGTCAGCCGCATCTTCTTT<br>R: AACTCTACTTTGCAGAACCTCAC	64.6 °C<br>64.6 °C	94
HPRT	3251	Hypoxanthine phosphoribosyltransferase 1	F: ACCAGTCAACAGGGGACATAA<br>R: CTTCGTGGGGTCCTTTTCACC	58.7 °C<br>61.2 °C	190
IFI44L	10964	Interferon induced protein 44 like	F: ACAGAGCCAAATGATTCCCTATG<br>R: TCGATAAACGACACACCAGTTG	58.6 °C<br>58.9 °C	124
IFITM1	8519	Interferon induced transmembrane protein 1	F: ACTCAACACTTCCTTCCCCAA<br>R: CTTCCTGTCCCTAGACTTCACG	59.2 °C<br>59.8 °C	231
SPRR1B	6699	Small proline rich protein 1B	F: TATTCCTCTCTTCACACCAG<br>R: TCCTTGGTTTTGGGGATG	53.8 °C<br>54.3 °C	155

[a] F: Forward (5’→3’); R: Reverse (5’→3’). [b] Tm: Melting temperature.

Supplemental Data

Table S2. Summary of RNA-seq data including library construction, sequencing and mapping results.

Treatment	Sample	Library Size (Average)	Raw Reads	Clean Reads	Mapped Reads	Mapping Rate	GC%
Vehicle (DMSO)	1	588	12.526.531	11.931.359	10.537.310	88.3%	51
	2	696	14.816.823	14.044.340	12.334.470	87.8%	51
	3	680	14.596.730	13.858.108	12.218.747	88.2%	51
Avicequinone B	4	613	11.080.591	10.536.861	9.320.257	88.46%	50
	5	555	11.732.386	11.119.540	9.807.826	88.20%	50
	6	461	6.825.309	6.465.727	5.530.893	85.54%	50

Table S3. Differentially expressed genes in HT-29 cells treated with avicequinone B in comparison with control (DMSO-treated) counterparts.

Gene	Locus	Value 1 (Control)	Value 2 (Avicequinone B)	LogFC [a]	*p* value	*q* value
IFITM1	NC_000011.10:313990-315272	42.5409	2.42756	-4.13127	5.00E-05	0.00377623
LAPTM5	NC_000001.11:30732467-30757840	2.00973	0.201078	-3.32118	0.0001	0.00676519
IFI44L	NC_000001.11:78620381-78646145	7.62704	0.770775	-3.30674	5.00E-05	0.00377623
RSAD2	NC_000002.12:6828553-6898239	3.28666	0.460121	-2.83654	5.00E-05	0.00377623
EPSTI1	NC_000013.11:42886387-42992271	2.74703	0.431894	-2.66912	5.00E-05	0.00377623
TMEM40	NC_000003.12:12733529-12769457	1.25091	0.201787	-2.63207	5.00E-05	0.00377623
TNNC1	NC_000003.12:52451090-52454041	14.2123	2.47664	-2.52069	5.00E-05	0.00377623
GPRC5B	NC_000016.10:19858970-19886045	1.2985	0.232402	-2.48215	5.00E-05	0.00377623
CMPK2	NC_000002.12:6828553-6898239	8.46783	1.59231	-2.41087	5.00E-05	0.00377623
HOPX	NC_000004.12:56647987-56681866	2.20683	0.416391	-2.40596	5.00E-05	0.00377623
OASL	NC_000012.12:121018881-121039242	55.8964	10.7213	-2.38227	5.00E-05	0.00377623
CDX1	NC_000005.10:150166780-150184558	3.06173	0.616582	-2.31198	5.00E-05	0.00377623
XAF1	NC_000017.11:6755410-6775647	3.6127	0.732204	-2.30276	5.00E-05	0.00377623
HEPACAM2	NC_000007.14:93188585-93232297	4.04772	0.821486	-2.3008	5.00E-05	0.00377623
SGCA	NC_000017.11:50157836-50175932	1.10714	0.227868	-2.28057	0.00015	0.00930899
CIDEC	NC_000003.12:9866709-9880254	3.47535	0.716569	-2.27798	5.00E-05	0.00377623
PPP1R14D	NC_000015.10:40815444-40828793	13.2244	2.75664	-2.26222	5.00E-05	0.00377623
PIGR	NC_000001.11:206928521-206946466	7.01245	1.47269	-2.25146	5.00E-05	0.00377623
IFIT1	NC_000010.11:89392545-89406487	18.5847	3.94525	-2.23593	5.00E-05	0.00377623
SEPT4	NC_000017.11:58519836-58692055	1.53396	0.331877	-2.20854	5.00E-05	0.00377623
MX2	NC_000021.9:41304228-41408943	6.10635	1.32814	-2.2009	5.00E-05	0.00377623
CASP1	NC_000011.10:105025507-105035591	1.67499	0.384346	-2.12367	0.00085	0.0351483
LCE1E	NC_000001.11:152786276-152788426	2.82979	0.65109	-2.11977	0.00055	0.0257143
OAS2	NC_000012.12:112978394-	29.0267	6.94227	-2.0639	5.00E-05	0.00377623

Supplemental Data

	113011723					
IFI44	NC_000001.11:78648553-78664078	52.6196	12.7625	-2.0437	5.00E-05	0.00377623
AGXT	NC_000002.12:240868744-240879119	1.67463	0.410501	-2.02839	0.0011	0.0422045
CD200	NC_000003.12:112332223-112362812	5.58892	1.37295	-2.02529	5.00E-05	0.00377623
IFITM3	NC_000011.10:319672-320914	250.965	62.0231	-2.01661	5.00E-05	0.00377623
ISG15	NC_000001.11:1013466-1014540	594.444	148.447	-2.00159	5.00E-05	0.00377623
REG4	NC_000001.11:119794017-119811580	70.8985	17.7209	-2.0003	5.00E-05	0.00377623
RNASE1	NC_000014.9:20725951-20810034	3.88919	0.980171	-1.98837	5.00E-05	0.00377623
MAPK11	NC_000022.11:50263712-50270393	1.94488	0.492248	-1.98223	5.00E-05	0.00377623
IFI6	NC_000001.11:27666060-27672213	194.867	49.5202	-1.9764	5.00E-05	0.00377623
PLLP	NC_000016.10:57256096-57284672	1.80507	0.459813	-1.97294	0.001	0.039516
NOTUM	NC_000017.11:81952506-81961181	1.7484	0.446327	-1.96986	0.00015	0.00930899
MX1	NC_000021.9:41420328-41459214	33.7418	8.68634	-1.95772	5.00E-05	0.00377623
IFIT2	NC_000010.11:89293472-89316145	14.1052	3.63721	-1.95532	5.00E-05	0.00377623
DDX60	NC_000004.12:168216290-168318807	9.93407	2.586	-1.94166	5.00E-05	0.00377623
PGF	NC_000014.9:74941829-74955764	1.53897	0.405425	-1.92446	0.00035	0.018209
APOE	NC_000019.10:44905748-44909395	6.08789	1.6106	-1.91834	5.00E-05	0.00377623
NTF4	NC_000019.10:49054273-49065076	3.65749	0.979458	-1.9008	0.0001	0.00676519
PAX4	NC_000007.14:127610291-127618192	1.33869	0.364996	-1.87486	0.00125	0.0466054
PTPRO	NC_000012.12:15322256-15598331	7.26995	2.0539	-1.82358	5.00E-05	0.00377623
LCK	NC_000001.11:32251238-32286167	1.17102	0.337414	-1.79517	0.0003	0.0161565
HERC6	NC_000004.12:88378564-88506170	13.2967	3.85989	-1.78443	5.00E-05	0.00377623
IFIT3	NC_000010.11:89327818-89344944	32.7464	9.56394	-1.77566	5.00E-05	0.00377623
GUCA2A	NC_000001.11:42162690-42164724	5.79368	1.69985	-1.76907	0.00115	0.0436419
ANXA13	NC_000008.11:123680793-123766246	66.1225	19.4665	-1.76415	5.00E-05	0.00377623
NEUROG3	NC_000010.11:69571439-69577099	3.04965	0.919591	-1.72958	0.0004	0.020233
GJB6	NC_000013.11:20221961-20232395	1.30194	0.401172	-1.69836	0.00025	0.0139341
UBE2L6	NC_000011.10:57551654-57568330	17.2816	5.33834	-1.69477	5.00E-05	0.00377623
FFAR2	NC_000019.10:35447964-35451767	1.59041	0.495312	-1.68299	0.0005	0.023902
GBP1	NC_000001.11:89034665-89102691	1.9541	0.616409	-1.66455	0.00035	0.018209
SERPINE1	NC_000007.14:101127086-101139266	2.62158	0.830796	-1.65787	5.00E-05	0.00377623
OAS1	NC_000012.12:112906776-112932190	92.4562	29.3328	-1.65625	5.00E-05	0.00377623
KLK12	NC_000019.10:51028953-51035651	6.1343	1.95372	-1.65068	0.0001	0.00676519
IFI27	NC_000014.9:94110732-94116699	651.455	207.715	-1.64906	5.00E-05	0.00377623
NEURL1	NC_000010.11:103479602-103592552	3.07106	0.979787	-1.64819	5.00E-05	0.00377623
RARRES3	NC_000011.10:63536800-63546458	157.788	50.5314	-1.64273	5.00E-05	0.00377623
GPA33	NC_000001.11:167051126-167091553	2.43622	0.789404	-1.62581	5.00E-05	0.00377623

Supplemental Data

HYAL1	NC_000003.12:50299888-50312951	4.28803	1.41282	-1.60174	0.00015	0.00930899
NEURL3	NC_000002.12:96497642-96508157	2.99216	0.990892	-1.59439	5.00E-05	0.00377623
RHOV	NC_000015.10:40872213-40874289	6.57375	2.17994	-1.59243	5.00E-05	0.00377623
RARRES2	NC_000007.14:150338317-150341685	18.8919	6.34154	-1.57486	5.00E-05	0.00377623
COCH	NC_000014.9:30874495-30890618	2.93872	0.990655	-1.56874	5.00E-05	0.00377623
CLDN2	NC_000023.11:106900062-106930861	62.9028	21.3092	-1.56165	5.00E-05	0.00377623
FAM83C	NC_000020.11:35285250-35292422	1.13674	0.388841	-1.54766	0.00055	0.0257143
CLDN9	NC_000016.10:3012455-3014505	6.01097	2.05831	-1.54614	5.00E-05	0.00377623
ETNK2	NC_000001.11:204131060-204152182	1.2446	0.43096	-1.53005	0.0004	0.020233
GAMT	NC_000019.10:1397025-1401570	6.03035	2.0887	-1.52964	5.00E-05	0.00377623
WWTR1	NC_000003.12:149517234-149724783	1.18082	0.41277	-1.51639	5.00E-05	0.00377623
MAFB	NC_000020.11:40685847-40689240	1.64182	0.576004	-1.51114	0.0001	0.00676519
CARNS1	NC_000011.10:67415677-67425607	2.10177	0.74173	-1.50264	5.00E-05	0.00377623
AIF1L	NC_000009.12:131096475-131123152	2.78618	0.99164	-1.4904	5.00E-05	0.00377623
PSMB9	NC_000006.12:32854160-32859851	10.7217	3.8259	-1.48666	5.00E-05	0.00377623
S100A2	NC_000001.11:153561107-153565830	39.2015	14.097	-1.47552	5.00E-05	0.00377623
RBP4	NC_000010.11:93591835-93601744	12.4303	4.49303	-1.4681	5.00E-05	0.00377623
LGALS2	NC_000022.11:37570245-37580017	47.9498	17.3803	-1.46407	5.00E-05	0.00377623
PALM3	NC_000019.10:14053366-14062137	3.47439	1.26756	-1.4547	5.00E-05	0.00377623
ADH6	NC_000004.12:99088856-99301356	3.89607	1.43769	-1.43827	0.00015	0.00930899
LYPD6B	NC_000002.12:149038466-149215262	2.71453	1.00336	-1.43586	5.00E-05	0.00377623
SLC1A7	NC_000001.11:53087178-53142632	2.82452	1.04592	-1.43324	0.0001	0.00676519
SPHK1	NC_000017.11:76382976-76387860	2.1454	0.796574	-1.42937	0.0003	0.0161565
PDZK1IP1	NC_000001.11:47183588-47190099	67.8236	25.2021	-1.42824	5.00E-05	0.00377623
AQP7	NC_000009.12:33383134-33410302	1.20752	0.44907	-1.42704	0.00035	0.018209
IL1R1	NC_000002.12:102069637-102179874	1.22748	0.460053	-1.41583	0.0002	0.0117098
CX3CL1	NC_000016.10:57372460-57385048	2.39113	0.904247	-1.4029	5.00E-05	0.00377623
USP18	NC_000022.11:18149958-18177397	7.4884	2.85643	-1.39044	5.00E-05	0.00377623
RAET1L	NC_000006.12:150018333-150025596	5.96602	2.2771	-1.38957	5.00E-05	0.00377623
TFF3	NC_000021.9:42311666-42315596	25.0933	9.58104	-1.38904	5.00E-05	0.00377623
CEACAM7	NC_000019.10:41673306-41688277	1.88004	0.720893	-1.3829	0.0006	0.027416
CFB	NC_000006.12:31945943-31952084	10.9144	4.19933	-1.378	5.00E-05	0.00377623
FOLR1	NC_000011.10:72189557-72196323	15.7596	6.0742	-1.37547	5.00E-05	0.00377623
UPK3A	NC_000022.11:45284986-45295874	8.35012	3.22356	-1.37314	0.0003	0.0161565
BLVRA	NC_000007.14:43754840-43839020	6.18426	2.40321	-1.36363	0.00035	0.018209
KRT17	NC_000017.11:41619439-41624630	15.4625	6.07751	-1.34722	5.00E-05	0.00377623
COPZ2	NC_000017.11:48026166-48048091	4.32374	1.71784	-1.33168	0.0004	0.020233

Supplemental Data

PRAP1	NC_000010.11:133347339-133352683	34.2362	13.7289	-1.31831	5.00E-05	0.00377623
OAS3	NC_000012.12:112938432-112973251	59.775	24.0309	-1.31465	5.00E-05	0.00377623
TNFAIP8L3	NC_000015.10:51056595-51105276	7.44057	2.99874	-1.31106	5.00E-05	0.00377623
PLEKHA4	NC_000019.10:48837091-48868627	5.57248	2.25644	-1.30427	5.00E-05	0.00377623
CD74	NC_000005.10:150401636-150412936	12.0218	4.87479	-1.30224	5.00E-05	0.00377623
MAP1LC3A	NC_000020.11:34546822-34560345	26.0717	10.5958	-1.299	5.00E-05	0.00377623
SUSD3	NC_000009.12:93058687-93085138	3.74852	1.52781	-1.29486	0.0003	0.0161565
N4BP3	NC_000005.10:178113505-178127034	1.20235	0.4922	-1.28854	0.0001	0.00676519
TSPAN32	NC_000011.10:2296276-2318201	1.97298	0.813535	-1.2781	0.00075	0.0322677
PARP9	NC_000003.12:122525589-122575203	42.15	17.3886	-1.27739	5.00E-05	0.00377623
PRODH	NC_000022.11:18912773-18936553	1.88308	0.778253	-1.27478	0.0012	0.0452256
NES	NC_000001.11:156668762-156677397	1.70873	0.70665	-1.27386	0.00015	0.00930899
SLPI	NC_000020.11:45230820-45290352	10.3812	4.31824	-1.26546	0.0012	0.0452256
RASL11A	NC_000013.11:27270326-27273690	40.1973	16.7707	-1.26115	5.00E-05	0.00377623
SERPINA6	NC_000014.9:94304247-94323351	6.21789	2.5944	-1.26102	0.0001	0.00676519
C1orf115	NC_000001.11:220690285-220699157	2.02657	0.848347	-1.25632	0.0006	0.027416
CDX2	NC_000013.11:27960917-27969368	2.35487	1.00252	-1.23201	0.0004	0.020233
APOL3	NC_000022.11:36140322-36166177	3.36534	1.43891	-1.22578	5.00E-05	0.00377623
SECTM1	NC_000017.11:82321023-82334045	4.26709	1.83601	-1.21668	0.00045	0.0220627
SOWAHA	NC_000005.10:132813323-132816797	2.01462	0.875316	-1.20263	0.00025	0.0139341
SMPD1	NC_000011.10:6390300-6394998	4.85654	2.13595	-1.18505	5.00E-05	0.00377623
IFIH1	NC_000002.12:162267078-162318708	13.2086	5.83259	-1.17927	5.00E-05	0.00377623
UBA7	NC_000003.12:49805204-49813958	4.75	2.10834	-1.17182	5.00E-05	0.00377623
ST6GALNAC1	NC_000017.11:76617768-76643838	3.28893	1.46209	-1.16959	0.00015	0.00930899
LINC00261	NC_000020.11:22560552-22578642	9.96174	4.43652	-1.16697	5.00E-05	0.00377623
DHX58	NC_000017.11:42101403-42112733	9.02519	4.02228	-1.16595	5.00E-05	0.00377623
KCNH2	NC_000007.14:150944955-150978314	8.38604	3.74719	-1.16218	5.00E-05	0.00377623
FAM132B	NC_000002.12:238159007-238168890	2.28682	1.02864	-1.1526	0.0008	0.0338484
SCAMP5	NC_000015.10:74995534-75021495	4.23013	1.90644	-1.14982	0.0001	0.00676519
DDX58	NC_000009.12:32455301-32526324	10.5294	4.7583	-1.1459	5.00E-05	0.00377623
SLC5A9	NC_000001.11:48221112-48248644	1.163	0.526616	-1.14303	0.00135	0.048998
LOXL1	NC_000015.10:73917467-73952141	9.89426	4.49238	-1.13911	5.00E-05	0.00377623
HES6	NC_000002.12:238238266-238240124	36.1551	16.4592	-1.1353	5.00E-05	0.00377623
CIB2	NC_000015.10:78104605-78131976	4.29633	1.97181	-1.12358	0.0007	0.0307735
HES4	NC_000001.11:998961-1001052	13.2588	6.0935	-1.1216	5.00E-05	0.00377623
SERPINE2	NC_000002.12:223975047-224039319	5.12713	2.3618	-1.11826	5.00E-05	0.00377623
LY6E	NC_000008.11:143018484-143022410	326.82	151.639	-1.10786	5.00E-05	0.00377623

Supplemental Data

FAM131C	NC_000001.11:16057768-16073632	4.00417	1.85902	-1.10696	0.0011	0.0422045
FOXJ1	NC_000017.11:76136332-76240368	7.35402	3.4719	-1.08281	0.00015	0.00930899
MATN2	NC_000008.11:97869020-98036720	3.09348	1.46201	-1.08127	0.00025	0.0139341
SLC7A7	NC_000014.9:22773221-22819811	14.7377	6.99155	-1.07583	5.00E-05	0.00377623
AQP1	NC_000007.14:30911799-30925516	9.45705	4.48919	-1.07494	5.00E-05	0.00377623
AGR3	NC_000007.14:16854710-16881989	46.1377	21.9478	-1.07187	5.00E-05	0.00377623
ULBP2	NC_000006.12:149941937-149949235	10.7436	5.13179	-1.06594	5.00E-05	0.00377623
BST2	NC_000019.10:17402938-17405648	214.436	102.587	-1.06371	5.00E-05	0.00377623
CTSV	NC_000009.12:97029676-97039643	5.50947	2.63952	-1.06164	5.00E-05	0.00377623
VGLL1	NC_000023.11:136532151-136556807	7.4013	3.54918	-1.06029	0.00025	0.0139341
HLA-DMA	NC_000006.12:32948613-32953122	18.1999	8.73976	-1.05826	5.00E-05	0.00377623
CD6	NC_000011.10:60971640-61020377	1.55958	0.756242	-1.04424	0.00135	0.048998
TACSTD2	NC_000001.11:58575422-58577494	31.0952	15.2185	-1.03087	5.00E-05	0.00377623
TMPRSS4	NC_000011.10:118015771-118125505	42.9256	21.029	-1.02946	5.00E-05	0.00377623
SOD3	NC_000004.12:24795462-24800845	8.92351	4.37368	-1.02876	0.00015	0.009309
C2orf54	NC_000002.12:240886047-240896889	12.5282	6.16881	-1.02212	5.00E-05	0.00377623
TUBA4A	NC_000002.12:219245468-219272188	105.916	52.2283	-1.02002	5.00E-05	0.00377623
FBXO44	NC_000001.11:11648360-11663327	6.17793	3.05775	-1.01465	0.00095	0.0380109
MDK	NC_000011.10:46380783-46383837	116.157	57.8586	-1.00547	5.00E-05	0.00377623
HSH2D	NC_000019.10:16134027-16158575	7.62096	3.80238	-1.00307	0.00075	0.0322677
RAMP1	NC_000002.12:237858713-237912117	9.90607	4.95002	-1.00088	0.0006	0.027416
ADGRB2	NC_000001.11:31727104-31764063	2.15238	1.07688	-0.99908	0.0001	0.00676519
TUBB2A	NC_000006.12:3153665-3157549	19.0084	9.56792	-0.990361	5.00E-05	0.00377623
FLT3LG	NC_000019.10:49474171-49487038	5.88006	2.97305	-0.983887	0.0004	0.020233
NME4	NC_000016.10:382240-400754	56.7499	28.7151	-0.982807	5.00E-05	0.00377623
WNK4	NC_000017.11:42779700-42797066	11.1154	5.63265	-0.980673	5.00E-05	0.00377623
SARDH	NC_000009.12:133663073-133739958	1.88408	0.955597	-0.979385	0.0009	0.0363522
PLPPR2	NC_000019.10:11355385-11365698	6.07172	3.11608	-0.962372	5.00E-05	0.00377623
IFI35	NC_000017.11:43006724-43014459	38.3569	19.7291	-0.959157	5.00E-05	0.00377623
SP110	NC_000002.12:230165465-230316571	16.369	8.43213	-0.956997	5.00E-05	0.00377623
PDZK1	NC_000001.11:145607959-145707507	8.78657	4.53874	-0.953009	0.0003	0.0161565
IFIT5	NC_000010.11:89413855-89421506	6.26689	3.24151	-0.951085	5.00E-05	0.00377623
DPEP1	NC_000016.10:89613307-89641540	34.0581	17.6625	-0.947309	5.00E-05	0.00377623
IHH	NC_000002.12:219054419-219060516	16.0267	8.32306	-0.945295	5.00E-05	0.00377623
E2F2	NC_000001.11:23505695-23531266	3.33289	1.74016	-0.937555	0.0002	0.0117098
ZNF358	NC_000019.10:7513879-7534009	12.5802	6.59871	-0.930899	0.0007	0.0307735
NNMT	NC_000011.10:114295812-114312516	19.4155	10.1907	-0.929962	5.00E-05	0.00377623

Supplemental Data

HLA-F	NC_000006.12:29723339-29749049	10.4424	5.48805	-0.928089	0.00125	0.0466054
HMGCS2	NC_000001.11:119747995-119769125	86.5147	45.5703	-0.924851	5.00E-05	0.00377623
PLSCR1	NC_000003.12:146515177-146544841	93.6336	49.7704	-0.911739	5.00E-05	0.00377623
CDKN1C	NC_000011.10:2883217-2885804	7.77228	4.13937	-0.908927	0.0004	0.020233
CORO1A	NC_000016.10:30183392-30189076	6.55071	3.4904	-0.908258	0.00075	0.0322677
MELTF	NC_000003.12:197001739-197029816	15.3625	8.20154	-0.905443	5.00E-05	0.00377623
CDKN1A	NC_000006.12:36676459-36687339	30.2909	16.19	-0.903783	5.00E-05	0.00377623
APOL1	NC_000022.11:36253070-36267531	73.3644	39.2179	-0.903567	5.00E-05	0.00377623
CLIC3	NC_000009.12:136994607-136997929	53.3757	28.6522	-0.897539	5.00E-05	0.00377623
FAM3D	NC_000003.12:58505135-58706084	33.3341	17.9314	-0.894512	5.00E-05	0.00377623
P3H2	NC_000003.12:189956727-190144846	6.97843	3.75801	-0.892932	5.00E-05	0.00377623
RGS16	NC_000001.11:182598622-182604413	6.50438	3.50553	-0.891781	0.00025	0.0139341
PARM1	NC_000004.12:74933074-75080901	2.21097	1.19219	-0.891065	0.0005	0.023902
CA2	NC_000008.11:85438826-85481492	45.0742	24.3177	-0.890293	5.00E-05	0.00377623
MR1	NC_000001.11:181033378-181061938	1.25083	0.678419	-0.882638	0.0009	0.0363522
STAT1	NC_000002.12:190969035-191014250	71.1877	38.7303	-0.878165	5.00E-05	0.00377623
SELENBP1	NC_000001.11:151364301-151372734	43.1518	23.5697	-0.872491	5.00E-05	0.00377623
OR51E1	NC_000011.10:4643925-4655488	3.39203	1.85904	-0.86759	0.00085	0.0351483
PIK3AP1	NC_000010.11:96587099-96720522	8.43957	4.64407	-0.86178	5.00E-05	0.00377623
NOX1	NC_000023.11:100843323-100874345	25.875	14.2797	-0.857588	5.00E-05	0.00377623
C2orf72	NC_000002.12:231037565-231049714	4.6422	2.5632	-0.856863	0.00015	0.00930899
SAMHD1	NC_000020.11:36873070-36951843	16.6349	9.18573	-0.856746	5.00E-05	0.00377623
HLA-B	NC_000006.12:31353865-31357245	15.8849	8.77853	-0.855603	0.00015	0.00930899
SOSTDC1	NC_000007.14:16461480-16496513	8.23663	4.55802	-0.853646	0.0007	0.0307735
APOL2	NC_000022.11:36226208-36239954	31.3053	17.3461	-0.851799	5.00E-05	0.00377623
IRF7	NC_000011.10:612554-615999	51.6894	28.6415	-0.851761	5.00E-05	0.00377623
HPGD	NC_000004.12:174490176-174541049	11.6522	6.45688	-0.851697	5.00E-05	0.00377623
GGT1	NC_000022.11:24583749-24629005	15.1651	8.40349	-0.851697	5.00E-05	0.00377623
TAP1	NC_000006.12:32840716-32853971	15.0916	8.36626	-0.851093	0.001	0.039516
IL17RE	NC_000003.12:9902085-9916402	12.0954	6.71513	-0.848969	5.00E-05	0.00377623
PARP12	NC_000007.14:140023743-140063721	21.9367	12.1792	-0.848928	5.00E-05	0.00377623
TNFSF10	NC_000003.12:172505507-172523507	23.9149	13.3367	-0.842507	5.00E-05	0.00377623
PXDC1	NC_000006.12:3722601-3752012	19.6238	10.9834	-0.837283	5.00E-05	0.00377623
FAM105A	NC_000005.10:14581781-14616180	17.2444	9.67427	-0.833899	5.00E-05	0.00377623
HSPB1	NC_000007.14:76302557-76304297	377.485	211.792	-0.833773	5.00E-05	0.00377623
CDHR5	NC_000011.10:616564-625067	16.4447	9.25849	-0.828775	5.00E-05	0.00377623
CFH	NC_000001.11:196651877-196747504	16.5425	9.33914	-0.824819	0.0009	0.0363522

Supplemental Data

CDH3	NC_000016.10:68644819-68727573	4.72372	2.67536	-0.820193	0.0001	0.00676519
MYO7B	NC_000002.12:127535688-127637728	2.67765	1.51946	-0.81741	0.00035	0.018209
NBL1	NC_000001.11:19596976-19658456	44.5972	25.311	-0.817188	0.00065	0.0291442
AQP3	NC_000009.12:33441153-33447633	79.7312	45.2851	-0.816109	5.00E-05	0.00377623
MSLN	NC_000016.10:760745-768865	6.71097	3.82728	-0.810201	0.0006	0.027416
MYBL2	NC_000020.11:43667018-43716496	112.68	64.4847	-0.8052	5.00E-05	0.00377623
GGT6	NC_000017.11:4556926-4560596	15.0588	8.62062	-0.804743	5.00E-05	0.00377623
TMEM238	NC_000019.10:55379243-55384259	39.58	22.7952	-0.796038	0.00015	0.00930899
AOC1	NC_000007.14:150818963-150877309	36.0015	20.7941	-0.791881	5.00E-05	0.00377623
TCN2	NC_000022.11:30607082-30627060	26.2698	15.1893	-0.790352	5.00E-05	0.00377623
VAMP5	NC_000002.12:85584407-85593388	47.0174	27.2739	-0.785671	0.00015	0.00930899
DEGS2	NC_000014.9:100146415-100166886	46.9666	27.3271	-0.781302	5.00E-05	0.00377623
PPP1R1B	NC_000017.11:39626207-39636625	63.6854	37.2082	-0.775343	5.00E-05	0.00377623
TSPAN4	NC_000011.10:842823-867116	11.3134	6.62347	-0.772368	0.0009	0.0363522
NECTIN3	NC_000003.12:111071758-111201444	13.7388	8.0501	-0.771176	0.00035	0.018209
PLIN2	NC_000009.12:19108390-19127606	11.2308	6.60679	-0.765442	0.00025	0.0139341
IL15RA	NC_000010.11:5948896-5978741	6.50805	3.8383	-0.761759	0.0011	0.0422045
KRT13	NC_000017.11:41500980-41505613	13.0508	7.71907	-0.757636	0.0002	0.0117098
IGFBP2	NC_000002.12:216632827-216664436	21.7493	12.8844	-0.75534	0.00055	0.0257143
NINJ1	NC_000009.12:93121488-93134304	33.4916	19.8925	-0.751575	5.00E-05	0.00377623
SESN1	NC_000006.12:108848409-109094505	11.6179	6.90696	-0.750225	0.0001	0.00676519
ASAP3	NC_000001.11:23428562-23484631	3.44949	2.05104	-0.750028	0.00135	0.048998
ZG16B	NC_000016.10:2830171-2832284	24.1402	14.3692	-0.748456	0.00135	0.048998
CCL15	NC_000017.11:35983655-36059131	41.8381	24.9292	-0.746984	0.0001	0.00676519
LGALS4	NC_000019.10:38801670-38813544	1115.86	667.725	-0.740834	5.00E-05	0.00377623
RHOD	NC_000011.10:67056817-67072017	30.7428	18.4164	-0.739256	5.00E-05	0.00377623
IQGAP2	NC_000005.10:76403254-76708132	9.71864	5.82701	-0.737999	5.00E-05	0.00377623
NENF	NC_000001.11:212432886-212446379	37.8002	22.6877	-0.736483	0.0001	0.00676519
DDX60L	NC_000004.12:168356734-168480514	4.2996	2.58439	-0.734378	0.0001	0.00676519
FOXA3	NC_000019.10:45864259-45873797	13.6881	8.25434	-0.729693	0.00035	0.018209
CDH24	NC_000014.9:23047060-23058476	6.56938	3.97123	-0.726172	0.00075	0.0322677
KCNK5	NC_000006.12:39188970-39229475	16.8685	10.2061	-0.7249	5.00E-05	0.00377623
IRF9	NC_000014.9:24161212-24166565	29.1482	17.6628	-0.722695	0.0001	0.00676519
AZGP1	NC_000007.14:99966726-99976112	38.3646	23.3043	-0.719179	5.00E-05	0.00377623
HES1	NC_000003.12:194136141-194138612	37.3534	22.7126	-0.717742	5.00E-05	0.00377623
P2RX4	NC_000012.12:121121217-121234106	9.47461	5.76622	-0.71644	0.00085	0.0351483
CDR2L	NC_000017.11:74987631-75005800	9.92815	6.04242	-0.716399	5.00E-05	0.00377623

Supplemental Data

HELZ2	NC_000020.11:63558085-63574239	20.7858	12.6626	-0.715026	5.00E-05	0.00377623
CDC42EP5	NC_000019.10:54465024-54473290	27.8949	17.0294	-0.711971	0.00095	0.0380109
HEPH	NC_000023.11:66162525-66267389	15.1066	9.24692	-0.708138	5.00E-05	0.00377623
B2M	NC_000015.10:44711486-44718159	1159.84	711.132	-0.705739	5.00E-05	0.00377623
PARP10	NC_000008.11:143977151-143986471	15.095	9.25955	-0.705053	0.00015	0.00930899
LGALS9	NC_000017.11:27631147-27649560	44.9719	27.735	-0.697316	5.00E-05	0.00377623
PARP14	NC_000003.12:122680725-122730840	36.2684	22.389	-0.695922	5.00E-05	0.00377623
GJB1	NC_000023.11:71215211-71225215	71.0491	43.8628	-0.695818	5.00E-05	0.00377623
PRSS8	NC_000016.10:31131432-31135762	106.917	66.0117	-0.695703	5.00E-05	0.00377623
NRGN	NC_000011.10:124739932-124747206	28.5145	17.6193	-0.694542	0.00035	0.018209
C19orf25	NC_000019.10:1446267-1479552	15.3979	9.53075	-0.692071	0.00025	0.0139341
CLEC3A	NC_000016.10:78022545-78032106	89.462	55.5785	-0.686749	5.00E-05	0.00377623
EBP	NC_000023.11:48521775-48528716	131.17	81.9824	-0.678057	5.00E-05	0.00377623
FXYD3	NC_000019.10:35115820-35124324	110.639	69.4182	-0.672477	0.00045	0.0220627
C19orf66	NC_000019.10:10086118-10106194	14.8958	9.35096	-0.671715	0.0005	0.023902
ASF1B	NC_000019.10:14119508-14136628	77.6034	48.7236	-0.671499	5.00E-05	0.00377623
WNT10A	NC_000002.12:218859822-218893931	10.848	6.81986	-0.669621	0.00115	0.0436419
NEU1	NC_000006.12:31859051-31862932	76.9441	48.4477	-0.667383	5.00E-05	0.00377623
EPN3	NC_000017.11:50532686-50543750	10.7087	6.75049	-0.665715	0.0002	0.0117098
MGAT3	NC_000022.11:39447165-39492194	3.83881	2.42268	-0.664059	0.0011	0.0422045
NR4A1	NC_000012.12:52022831-52059507	11.3219	7.15013	-0.663076	0.0005	0.023902
MUC13	NC_000003.12:124905441-124934751	111.043	70.2736	-0.660059	5.00E-05	0.00377623
C12orf57	NC_000012.12:6943437-6946003	77.633	49.283	-0.655578	0.00025	0.0139341
CRABP2	NC_000001.11:156699605-156712900	532.611	338.947	-0.652023	5.00E-05	0.00377623
FBP1	NC_000009.12:94603132-94640257	96.7572	61.6478	-0.65032	5.00E-05	0.00377623
TGFBI	NC_000005.10:136021543-136063818	25.5914	16.3077	-0.650105	0.00015	0.00930899
NMB	NC_000015.10:84655128-84658611	50.0261	31.9028	-0.648996	0.00045	0.0220627
CRAT	NC_000009.12:129094793-129110791	36.0459	23.0017	-0.648096	5.00E-05	0.00377623
RAB26	NC_000016.10:2148143-2154165	16.509	10.5739	-0.642744	0.00085	0.0351483
WNT11	NC_000011.10:76186325-76210842	19.6365	12.6437	-0.635124	0.0008	0.0338484
RGS2	NC_000001.11:192809038-192812277	39.2711	25.2914	-0.63482	0.00015	0.00930899
BTG2	NC_000001.11:203305535-203309602	27.6493	17.8544	-0.630965	0.0001	0.00676519
PLD1	NC_000003.12:171600403-171810494	12.0577	7.79233	-0.629831	0.00105	0.0410261
DGAT2	NC_000011.10:75768732-75801536	14.7608	9.56564	-0.625836	0.00065	0.0291442
SLC10A3	NC_000023.11:154487305-154490690	42.7257	27.7344	-0.623426	5.00E-05	0.00377623
TPPP3	NC_000016.10:67389806-67393535	110.194	71.5791	-0.62244	5.00E-05	0.00377623
AQP5	NC_000012.12:49950740-49978444	40.009	26.0199	-0.620707	0.0006	0.027416

Supplemental Data

LGALS3	NC_000014.9:55129216-55145430	78.0749	50.8697	-0.618054	5.00E-05	0.00377623
LAP3	NC_000004.12:17577303-17607967	65.5759	42.7441	-0.617441	5.00E-05	0.00377623
PSME2	NC_000014.9:24143364-24160661	171.49	111.93	-0.615528	0.0009	0.0363522
EFNA1	NC_000001.11:155127872-155134910	27.9665	18.2621	-0.614848	0.00075	0.0322677
BGN	NC_000023.11:153447664-153509554	48.1792	31.4837	-0.613807	0.00045	0.0220627
E2F1	NC_000020.11:33675485-33686404	37.0006	24.1823	-0.613594	0.00025	0.0139341
RAP1GAP	NC_000001.11:21596214-21669444	57.1539	37.3666	-0.613104	5.00E-05	0.00377623
TCEAL9	NC_000023.11:103356451-103358469	51.7985	33.9291	-0.61039	0.00035	0.018209
REEP6	NC_000019.10:1491165-1497925	73.1266	48.1003	-0.60435	0.0001	0.00676519
FUCA1	NC_000001.11:23845076-23868369	44.7016	29.4179	-0.603632	0.0002	0.0117098
ACOT11	NC_000001.11:54548133-54634744	12.3771	8.1502	-0.602766	0.0008	0.0338484
RMI2	NC_000016.10:11249004-11523825	38.3233	25.2384	-0.602601	0.00095	0.0380109
MCAM	NC_000011.10:119308523-119317132	22.3692	14.7325	-0.602509	0.0002	0.0117098
SYT7	NC_000011.10:61513715-61588404	15.0129	9.89807	-0.600983	0.0002	0.0117098
IFITM2	NC_000011.10:308106-309410	110.472	72.8402	-0.600875	0.0002	0.0117098
CCND3	NC_000006.12:41934932-42048894	58.0808	38.5237	-0.592314	0.0001	0.00676519
FCGRT	NC_000019.10:49512278-49526428	112.03	74.3203	-0.592062	0.0002	0.0117098
QPRT	NC_000016.10:29670587-29698699	46.8248	31.0655	-0.591963	0.00025	0.0139341
MAPK3	NC_000016.10:30114104-30123309	43.8144	29.0984	-0.590465	0.0006	0.027416
CALM3	NC_000019.10:46601254-46625118	301.256	200.09	-0.590341	5.00E-05	0.00377623
TUBA1B	NC_000012.12:49127781-49131521	722.269	480.143	-0.589071	0.0003	0.0161565
PXMP2	NC_000012.12:132687605-132704991	51.6483	34.3554	-0.588185	0.00085	0.0351483
TDRD7	NC_000009.12:97412019-97496125	13.8804	9.25517	-0.584716	0.0011	0.0422045
CENPM	NC_000022.11:41927746-41947164	41.9827	27.9988	-0.584431	0.00135	0.048998
KLHDC7A	NC_000001.11:18480929-18485999	8.67603	5.79118	-0.583178	0.0007	0.0307735
CPTP	NC_000001.11:1324755-1328896	25.129	16.7791	-0.582686	0.00035	0.018209
LMO4	NC_000001.11:87328467-87348923	32.3606	21.6108	-0.582488	0.0001	0.00676519
HOXA11-AS	NC_000007.14:27185407-27189293	32.2607	21.5443	-0.582473	0.00105	0.0410261
TRIM21	NC_000011.10:4384896-4393696	22.3151	14.9514	-0.577742	0.0012	0.0452256
ACSF2	NC_000017.11:50426157-50474845	43.4267	29.1343	-0.575861	0.00025	0.0139341
MTSS1L	NC_000016.10:70661203-70686207	12.5524	8.42574	-0.575091	0.0003	0.0161565
NPDC1	NC_000009.12:137039456-137046224	63.4853	42.7274	-0.57126	0.00015	0.00930899
CST3	NC_000020.11:23627896-23638048	294.995	199.255	-0.566076	5.00E-05	0.00377623
ATP1B1	NC_000001.11:169106708-169462221	297.791	201.201	-0.565659	0.00035	0.018209
ATP1B3	NC_000003.12:141876627-141926540	70.0037	47.479	-0.56014	5.00E-05	0.00377623
FEN1	NC_000011.10:61792636-61797244	102.107	69.3154	-0.558831	0.0002	0.0117098
NPC2	NC_000014.9:74479939-74493475	101.245	68.7504	-0.558415	0.00055	0.0257143

Supplemental Data

HLA-C	NC_000006.12:31268748-31272136	57.051	38.9024	-0.552393	0.00085	0.0351483
ID2	NC_000002.12:8681982-8684453	60.2716	41.1031	-0.55223	0.0005	0.023902
IL22RA1	NC_000001.11:24119770-24143285	27.4222	18.7548	-0.548089	0.0005	0.023902
CD81	NC_000011.10:2328748-2397419	254.252	173.937	-0.547693	0.0004	0.020233
RTN4R	NC_000022.11:20241414-20268293	30.2838	20.7271	-0.547026	0.0013	0.0479098
CYSTM1	NC_000005.10:140175067-140243789	278.611	190.757	-0.546511	5.00E-05	0.00377623
SP6	NC_000017.11:47844907-47876312	23.016	15.7918	-0.543456	0.00035	0.018209
RPS9P2	NC_000022.11:41063995-41064705	178.373	122.41	-0.543176	0.0005	0.023902
S100A14	NC_000001.11:153614254-153616917	146.946	101.1	-0.539508	0.00055	0.0257143
SEPW1	NC_000019.10:47778584-47784682	186.75	129.304	-0.530344	0.00045	0.0220627
WFS1	NC_000004.12:6260367-6308709	41.9984	29.0882	-0.529901	0.0004	0.020233
OAZ2	NC_000015.10:64687573-64703281	71.0834	49.3067	-0.527728	0.00045	0.0220627
FGFR3	NC_000004.12:1793298-1808872	21.453	14.8921	-0.526632	0.0011	0.0422045
ITM2C	NC_000002.12:230864597-230879254	133.684	93.0923	-0.522091	0.00065	0.0291442
PRR15L	NC_000017.11:47951966-47957905	50.8797	35.4701	-0.520488	0.00135	0.048998
AGR2	NC_000007.14:16791810-16805114	587.194	410.12	-0.517793	0.00055	0.0257143
IDI1	NC_000010.11:1018906-1056716	70.2326	49.2373	-0.51239	0.00045	0.0220627
DHCR24	NC_000001.11:54849626-54887248	211.115	148.296	-0.509546	0.0007	0.0307735
GUSB	NC_000007.14:65960683-65982314	55.8062	39.217	-0.508946	0.001	0.039516
VWA1	NC_000001.11:1430156-1442882	56.4819	39.7626	-0.506377	0.0004	0.020233
TMEM54	NC_000001.11:32885964-32901435	323.199	227.583	-0.506033	0.0004	0.020233
SLBP	NC_000004.12:1692730-1712741	57.0828	40.297	-0.502384	0.00085	0.0351483
FTH1P20	NC_000002.12:180692103-180916939	213.278	302.447	0.503945	0.00075	0.0322677
ND1	NC_012920.1:3306-4262	543.642	771.45	0.504914	0.0008	0.0338484
HK2	NC_000002.12:74832654-74893354	14.3402	20.3678	0.506223	0.0011	0.0422045
ATP9A	NC_000020.11:51596774-51768411	35.3742	50.3027	0.50794	0.0003	0.0161565
TEAD1	NC_000011.10:12674421-12944737	13.1709	18.7602	0.510322	0.00025	0.0139341
MED13	NC_000017.11:61942604-62065983	11.2171	15.9787	0.510442	0.0007	0.0307735
BBX	NC_000003.12:107522935-107811329	11.1661	15.9278	0.512419	0.00085	0.0351483
FAM208B	NC_000010.11:5684758-5763740	9.0796	12.9872	0.516385	0.00075	0.0322677
HMGB3	NC_000023.11:150980507-150990775	19.3756	27.726	0.516998	0.00085	0.0351483
FTH1P10	NC_000005.10:17353712-17354631	262.302	375.543	0.517753	0.00015	0.00930899
NTSR1	NC_000020.11:62708769-62762771	36.9341	52.9946	0.52089	0.0009	0.0363522
NF1	NC_000017.11:31094926-31377677	14.7526	21.228	0.524999	0.0004	0.020233
MUC5AC	NC_000011.10:1157952-1201138	3.02008	4.3591	0.529442	0.0011	0.0422045
RICTOR	NC_000005.10:38845857-39074421	4.80669	6.95436	0.532873	0.0008	0.0338484
KIAA0430	NC_000016.10:15434467-15643166	5.68016	8.22526	0.534129	0.0013	0.0479098

Supplemental Data

SOCS7	NC_000017.11:38351875-38405593	9.3671	13.5713	0.534885	0.0007	0.0307735
CHD9	NC_000016.10:53054990-53327502	3.07985	4.46502	0.535808	0.00105	0.0410261
EGFR	NC_000007.14:55019031-55207338	6.46922	9.4279	0.543345	0.001	0.039516
MAN1A2	NC_000001.11:117367392-117528872	8.24394	12.0274	0.544921	0.00125	0.0466054
TAOK1	NC_000017.11:29390924-29551904	7.01893	10.248	0.546019	0.0003	0.0161565
ABLIM3	NC_000005.10:149141482-149260439	15.4052	22.5027	0.546674	0.00025	0.0139341
DSP	NC_000006.12:7540113-7586717	12.0974	17.6837	0.54772	0.00025	0.0139341
SRRM2	NC_000016.10:2737075-2771412	39.8305	58.2348	0.54801	0.0006	0.027416
ND2	NC_012920.1:4469-5511	469.47	686.78	0.548816	0.00035	0.018209
CAD	NC_000002.12:27217256-27243792	11.9642	17.5029	0.548867	0.00025	0.0139341
HIPK2	NC_000007.14:139561569-139777894	3.68809	5.39885	0.549777	0.00035	0.018209
CDK6	NC_000007.14:92604920-92917187	6.81181	10.0217	0.557011	0.0002	0.0117098
ZNF587	NC_000019.10:57849812-57865123	8.57899	12.627	0.557638	0.00045	0.0220627
FKTN	NC_000009.12:105554034-105655950	3.40622	5.01776	0.558873	0.00075	0.0322677
SLC3A2	NC_000011.10:62856011-62888883	90.8293	133.857	0.559459	5.00E-05	0.00377623
XPOT	NC_000012.12:64404349-64451125	32.537	48.0456	0.562322	0.0001	0.00676519
H1F0	NC_000022.11:37805106-37807436	133.092	197.127	0.5667	0.0002	0.0117098
FAT1	NC_000004.12:186587782-186726915	21.5312	31.9228	0.568163	5.00E-05	0.00377623
MACF1	NC_000001.11:39084166-39487138	7.79624	11.5593	0.568199	0.00075	0.0322677
BNIP2	NC_000015.10:59659145-59689534	8.46531	12.5634	0.569595	0.00035	0.018209
TIPARP	NC_000003.12:156673170-156706770	21.6056	32.1724	0.574423	0.0001	0.00676519
ZFC3H1	NC_000012.12:71609598-71680648	5.12783	7.64805	0.576742	0.00065	0.0291442
MACC1	NC_000007.14:19918980-20217390	3.83359	5.71933	0.577151	0.00055	0.0257143
PPP1R15A	NC_000019.10:48872391-48876062	28.2211	42.1381	0.578353	0.00015	0.00930899
ARID2	NC_000012.12:45729682-45908037	3.50095	5.23995	0.581808	0.0009	0.0363522
TTC14	NC_000003.12:180481502-180679495	8.21289	12.3046	0.583234	0.00135	0.048998
ATRX	NC_000023.11:77504877-77786269	7.5539	11.3205	0.583643	5.00E-05	0.00377623
KIAA1109	NC_000004.12:122152332-122362759	3.99599	5.99238	0.584573	0.0002	0.0117098
HECTD1	NC_000014.9:31100114-31208060	14.3138	21.4726	0.585098	0.00015	0.00930899
ANXA3	NC_000004.12:78551587-78610451	67.327	101.311	0.589534	5.00E-05	0.00377623
EMP1	NC_000012.12:13196667-13216774	9.10301	13.7109	0.590907	0.0009	0.0363522
PRKDC	NC_000008.11:47773107-47960183	18.2133	27.4753	0.593141	5.00E-05	0.00377623
SEL1L	NC_000014.9:81471546-81533864	8.2174	12.4011	0.593716	0.0011	0.0422045
DUSP4	NC_000008.11:29333061-29350750	4.94418	7.46468	0.594348	0.0008	0.0338484
GLTSCR1L	NC_000006.12:42746957-42868560	3.65014	5.52861	0.598964	0.00135	0.048998
PRRC2C	NC_000001.11:171485495-171593511	11.8641	17.9816	0.599924	5.00E-05	0.00377623
PHIP	NC_000006.12:78867485-79078294	3.23961	4.91272	0.6007	0.00105	0.0410261

Supplemental Data

MYSM1	NC_000001.11:58654738-58700091	3.21197	4.88497	0.60489	0.0007	0.0307735
KIF14	NC_000001.11:200551496-200620791	3.84757	5.86186	0.60741	0.0005	0.023902
SGO2	NC_000002.12:200510197-200584095	4.74619	7.23828	0.608875	0.0008	0.0338484
EP400	NC_000012.12:131949919-132080466	3.67189	5.60927	0.611288	0.00015	0.00930899
LTN1	NC_000021.9:28928143-28992959	3.40292	5.22155	0.617703	0.00075	0.0322677
DST	NC_000006.12:56457986-56954671	4.32121	6.63407	0.61846	0.0001	0.00676519
AREG	NC_000004.12:74445097-74455009	94.8225	145.63	0.619006	5.00E-05	0.00377623
GCLM	NC_000001.11:93885198-93909598	7.56234	11.6265	0.62051	0.0003	0.0161565
ARFGEF3	NC_000006.12:138161689-138344663	3.27698	5.0413	0.62143	0.0001	0.00676519
ATXN1	NC_000006.12:16299111-16766883	4.16112	6.41068	0.623504	0.00065	0.0291442
NRIP1	NC_000021.9:14961234-15065903	9.68031	14.9273	0.624829	5.00E-05	0.00377623
CENPF	NC_000001.11:214603180-214664574	13.6763	21.11	0.626247	5.00E-05	0.00377623
SLC7A5	NC_000016.10:87830021-87869499	40.1707	62.0546	0.627393	5.00E-05	0.00377623
FLNA	NC_000023.11:154348528-154374638	47.1481	73.0588	0.63186	5.00E-05	0.00377623
LRP8	NC_000001.11:53226891-53328149	42.7676	66.4028	0.634725	0.00125	0.0466054
ABHD2	NC_000015.10:89088149-89202360	51.525	80.1365	0.637185	5.00E-05	0.00377623
ANXA10	NC_000004.12:168092524-168199047	71.0867	110.713	0.639167	5.00E-05	0.00377623
MIB1	NC_000018.10:21740792-21870957	5.28548	8.24955	0.642282	0.00075	0.0322677
GCLC	NC_000006.12:53497340-53545129	19.5468	30.5676	0.645068	5.00E-05	0.00377623
CEACAM6	NC_000019.10:41755420-41772211	8.10616	12.6793	0.64538	0.0011	0.0422045
ZNF292	NC_000006.12:87155550-87264172	2.45173	3.84993	0.651032	0.0003	0.0161565
CAPN8	NC_000001.11:223541608-223665734	17.2319	27.0913	0.652742	0.0002	0.0117098
BTAF1	NC_000010.11:91923768-92030998	5.17844	8.1431	0.653061	0.00015	0.00930899
ZBTB41	NC_000001.11:197153681-197200542	2.40056	3.77611	0.653532	0.00045	0.0220627
AKAP11	NC_000013.11:42271469-42323267	6.15062	9.6827	0.654678	5.00E-05	0.00377623
TBC1D5	NC_000003.12:17157161-17742739	3.67817	5.80004	0.657074	0.00055	0.0257143
RBM33	NC_000007.14:155644493-155781485	6.72548	10.6139	0.658242	5.00E-05	0.00377623
FGF19	NC_000011.10:69698237-69704338	29.6987	46.8797	0.658564	5.00E-05	0.00377623
ADAM19	NC_000005.10:157477303-157575823	3.73145	5.90408	0.661979	0.00025	0.0139341
QSER1	NC_000011.10:32892825-33015362	7.86245	12.4662	0.664972	5.00E-05	0.00377623
ANKRD12	NC_000018.10:9136752-9285985	3.2695	5.19173	0.667147	0.00045	0.0220627
VPS13D	NC_000001.11:12230038-12512047	1.86621	2.96495	0.667898	5.00E-05	0.00377623
CEP350	NC_000001.11:179954772-180114880	2.51543	4.00228	0.670016	5.00E-05	0.00377623
BRCA2	NC_000013.11:32315479-32399672	2.37144	3.77492	0.67068	0.0003	0.0161565
ABCG1	NC_000021.9:42199688-42304389	6.89775	10.9897	0.671958	0.0009	0.0363522
DEPDC1	NC_000001.11:68474151-68538627	6.44705	10.3004	0.675994	0.00015	0.00930899
ZCCHC11	NC_000001.11:52423274-52553463	3.84783	6.1547	0.677643	0.0003	0.0161565

Supplemental Data

DGKH	NC_000013.11:42040035-42256584	3.02129	4.83456	0.678219	0.0001	0.00676519
MTX3	NC_000005.10:79976715-79991267	1.87527	3.00513	0.680327	0.00075	0.0322677
BDP1	NC_000005.10:71455614-71578288	3.24263	5.20582	0.682962	5.00E-05	0.00377623
SMG1	NC_000016.10:18804852-18937762	7.03782	11.3111	0.684539	5.00E-05	0.00377623
VPS13A	NC_000009.12:77176755-77421537	3.4499	5.57324	0.691959	5.00E-05	0.00377623
ANAPC1	NC_000002.12:111766146-111884196	7.34977	11.9076	0.696118	0.00095	0.0380109
SLC38A1	NC_000012.12:46183054-46278613	31.7006	51.5983	0.702813	5.00E-05	0.00377623
CYP1B1	NC_000002.12:37920788-38076181	7.60455	12.4111	0.706699	5.00E-05	0.00377623
UVSSA	NC_000004.12:1345661-1388049	4.0506	6.63224	0.711361	0.0008	0.0338484
UTP20	NC_000012.12:101280126-101386619	2.66149	4.41517	0.730231	0.0001	0.00676519
CASC5	NC_000015.10:40594011-40664342	4.25433	7.05866	0.730461	5.00E-05	0.00377623
TTBK2	NC_000015.10:42738973-42920995	1.77098	2.94894	0.735647	0.0002	0.0117098
BRWD3	NC_000023.11:80669487-80809736	3.3231	5.5459	0.738892	0.0013	0.0479098
LOC105369370	NC_000011.10:69366824-69372512	2.57017	4.30615	0.744538	0.00025	0.0139341
CALB2	NC_000016.10:71358712-71392129	48.0807	80.7608	0.748198	5.00E-05	0.00377623
DCBLD2	NC_000003.12:98795969-98901689	43.0967	72.692	0.75422	5.00E-05	0.00377623
PHLDA1	NC_000012.12:76025446-76031776	21.4561	36.3713	0.761408	5.00E-05	0.00377623
LPP	NC_000003.12:188151205-188890671	3.1205	5.37336	0.784046	5.00E-05	0.00377623
ATF3	NC_000001.11:212565333-212620777	7.15249	12.3503	0.788026	5.00E-05	0.00377623
ZKSCAN8	NC_000006.12:28141643-28159472	2.65664	4.59228	0.789611	5.00E-05	0.00377623
ZNF532	NC_000018.10:58862599-58986480	4.00508	6.99848	0.805211	5.00E-05	0.00377623
SNX29	NC_000016.10:11976733-12574289	1.45527	2.54791	0.808025	0.0003	0.0161565
TMTC2	NC_000012.12:82686951-83134868	6.91152	12.1585	0.814891	5.00E-05	0.00377623
CENPE	NC_000004.12:103105805-103198409	3.82639	6.764	0.821893	5.00E-05	0.00377623
GAS2L3	NC_000012.12:100573660-100628288	2.82832	5.06214	0.839802	0.00085	0.0351483
CREB3L2	NC_000007.14:137874978-138002101	3.01024	5.43189	0.851575	0.00015	0.00930899
REL	NC_000002.12:60881494-60928171	1.31429	2.37967	0.85648	0.0002	0.0117098
CREB5	NC_000007.14:28299320-28825894	0.854062	1.55096	0.860748	0.00135	0.048998
LGR6	NC_000001.11:202193989-202319761	1.62151	2.97004	0.873147	0.001	0.039516
SORBS1	NC_000010.11:95311772-95561420	0.737856	1.36492	0.887403	5.00E-05	0.00377623
PSD3	NC_000008.11:18527302-19086909	1.47484	2.73411	0.890518	5.00E-05	0.00377623
ASPM	NC_000001.11:197084126-197146694	6.49836	12.1561	0.903533	5.00E-05	0.00377623
NEAT1	NC_000011.10:65422797-65445540	49.8066	94.0846	0.917622	5.00E-05	0.00377623
CLIP4	NC_000002.12:29097675-29183813	1.12211	2.1577	0.943287	0.00065	0.0291442
XIST	NC_000023.11:73792204-73852753	4.01727	7.8923	0.97423	5.00E-05	0.00377623
FAM129A	NC_000001.11:184791024-184975132	2.2605	4.45162	0.977689	5.00E-05	0.00377623
EREG	NC_000004.12:74365142-74388760	14.0173	27.6727	0.981257	5.00E-05	0.00377623

Supplemental Data

ADGRF1	NC_000006.12:46997702-47042363	5.7863	11.4912	0.989821	5.00E-05	0.00377623
IGSF9B	NC_000011.10:133908563-133956960	0.750953	1.50213	1.00021	0.0011	0.0422045
PSAT1	NC_000009.12:78297074-78330093	33.8886	70.913	1.06525	5.00E-05	0.00377623
TTLL7	NC_000001.11:83861110-83999340	0.730706	1.53107	1.06718	0.0012	0.0452256
TTC6	NC_000014.9:37595198-37842628	0.602631	1.26312	1.06764	0.0007	0.0307735
ABCA1	NC_000009.12:104781001-104939096	0.59561	1.27292	1.0957	5.00E-05	0.00377623
LOC400706	NC_000019.10:46057677-46077629	1.72067	3.68172	1.09741	0.0001	0.00676519
CHAC1	NC_000015.10:40942134-40956519	4.4221	9.46476	1.09783	5.00E-05	0.00377623
CEMIP	NC_000015.10:80779342-80989878	13.475	29.0212	1.10683	0.00055	0.0257143
CTH	NC_000001.11:70411217-70441949	2.07747	4.54347	1.12896	0.0002	0.0117098
PTPRM	NC_000018.10:7567315-8406861	0.75628	1.71462	1.18089	5.00E-05	0.00377623
SLCO5A1	NC_000008.11:69667045-69837829	0.505013	1.17902	1.22319	0.00085	0.0351483
PGM5	NC_000009.12:68355163-68531061	1.17393	2.76146	1.23409	0.0004	0.020233
TNC	NC_000009.12:115019574-115118257	0.940044	2.27738	1.27658	5.00E-05	0.00377623
SERPINA3	NC_000014.9:94612376-94624053	1.57644	4.09036	1.37556	0.0002	0.0117098
LRRN4	NC_000020.11:6040545-6054080	0.530669	1.39522	1.39461	0.00075	0.0322677
SLC6A9	NC_000001.11:43991246-44040680	0.799768	2.18878	1.45248	0.00115	0.0436419
ASNS	NC_000007.14:97852116-97872542	26.8663	89.2487	1.73203	5.00E-05	0.00377623
SCEL	NC_000013.11:77535673-77645263	2.62774	8.72945	1.73207	5.00E-05	0.00377623
CYP1A1	NC_000015.10:74719541-74725610	0.523368	1.76512	1.75387	5.00E-05	0.00377623
SLC7A11	NC_000004.12:138025237-138242418	5.73686	19.9331	1.79684	5.00E-05	0.00377623
AKAP12	NC_000006.12:151239814-151358559	1.51284	5.72766	1.92069	5.00E-05	0.00377623
SPRR1B	NC_000001.11:153031202-153032900	1.87231	8.19831	2.13051	5.00E-05	0.00377623

[a] LogFC: Log2 of Fold Change

Supplemental Data

Table S4. Top ten differentially expressed genes (Down-regulated) by Avicequinone B

Gene Symbol	Description	Function	Association with CRC	LogFC	*q* value
IFITM1	Interferon-induced transmembrane protein 1	IFN-induced antiviral protein	↑ Expression in CRC tissue is associated with an aggressive phenotype and poor prognosis.<br>↑ Proliferation and invasiveness of CRC cells (SW480 and DLD-1) with stable/ectopic over-expression of IFITM1.<br>↓ Migration/invasion ability of CRC cells (HT-29, LoVo, and DLD-1) with transient/stable knockdown of IFITIM1. [6-9]	-4.131	0.004
LAPTM5	Lysosomal-associated transmembrane protein 5	May have a special functional role during embryogenesis and in adult hematopoietic cells.	Predicted target of hsa-miR-150, hsa-miR-16 and hsa-miR-28, miRNAs down-regulated in CRC tissue.[10]	-3.321	0.007
IFI44L	Interferon-induced protein 44-like	Exhibits a low antiviral activity against hepatitis C virus.	↑ Expression in KRAS-mutated CRC cell lines and patient-derived organoid culture.<br>↑ Expression in HT-29 cells resistant to Oxaliplatin.[11,12]	-3.307	0.004
RSAD2	Radical S-adenosyl methionine domain-containing protein 2	Interferon-inducible iron-sulfur (4FE-4S) cluster-binding antiviral protein.	↑ Expression in CRC samples when compared to colorectal adenomas.[13]	-2.836	0.004
EPSTI1	Epithelial-stromal interaction protein 1	-----	↑ Expression in metastatic CRC cell lines compared to primary CRC cells.<br>↑ Expression in CRC cell lines with silenced expression of Nkx2-3, a transcription factor down regulated in CRC.[14,15]	-2.669	0.004
TMEM40	Transmembrane protein 40	-----	-----	-2.632	0.004
TNNC1	Troponin C (TN-C)	Troponin is the central regulatory protein of striated muscle contraction.	↑ Expression in CRC cells (SW480) treated with the ligand of CXCR4 and CXCR7, chemokines receptors increased with tumor stage and size in CRC.[16]	-2.521	0.004
GPRC5B	G-protein coupled receptor family C group 5 member B	Unknown	Targeted by hsa-miR-99b-5p, a miRNA down-regulated in CRC tissue.[17]	-2.482	0.004
CMPK2	UMP-CMP kinase 2, mitochondrial (cytidine/uridine monophosphate kinase 2)	May participate in dUTP and dCTP synthesis in mitochondria.	↑ mRNA expression is correlated to a higher risk score in stages I-III CRC, DNA aneuploidy, and neoplastic progression.[18,19]	-2.411	0.004
HOPX	Homeodomain-only protein	Atypical homeodomain protein which is required to modulate cardiac growth and development.	Colon stem cell marker. ↓ Expression in CRC tissue, mainly in an epigenetic manner, which is associated with increased progression and metastasis. Forced expression of HOPX suppressed proliferation and invasion of CRC cells (HCT116 and DLD1).[20]	-2.406	0.004

Supplemental data

Table S5. Top ten differentially expressed genes (Up-regulated) by Avicequinone B

Gene Symbol	Description	Function	Association with CRC	LogFC	*q* value
SPRR1B	Cornifin-B	Cross-linked envelope protein of keratinocytes.	↑ mRNA expression in HT-29 cells with knockdown expression of PHGR1, a possible protein marker of CRC development and metastasis.[21]	2.131	0.004
AKAP12	A-kinase anchor protein 12 (Gravin)	Anchoring protein that mediates the subcellular compartmentation of protein kinase A and C.	↓ mRNA expression or methylation of gene promoter in CRC tissue.<br>► Tumor suppressor gene in CRC. Represents a potential molecular biomarker for predicting CRC malignancy.<br>► Induced re-expression of AKAP12 promoted apoptosis, reduced colony formation and migration of LoVo cells *in vitro* and *in vivo*.[22-24]	1.921	0.004
SLC7A11	Cystine/glutamate transporter (xCT)	High affinity exchange of anionic aminoacids.	↓ Protein expression in metastatic CRC cells.[25]	1.797	0.004
CYP1A1	Cytochrome P450 1A1	Heme-thiolate monooxygenase that oxidizes a variety of compounds, including xenobiotics.	► Polymorphisms in CYP1A1 gene are associated with increased risk of CRC development.<br>► Key metabolizing enzyme of polycyclic aromatic hydrocarbons (i.e. benzo[a]pyrene) which are considered important carcinogens.[26-28]	1.754	0.004
SCEL	Sciellin	May function in the assembly or regulation of proteins in the cornified envelope.	↓ Expression in specimens from CRC patients with metastasis.<br>↓ Expression in CRC metastatic cell lines promoted migration and invasion, while overexpression had the opposite effect.[29]	1.732	0.004
ASNS	Asparagine synthetase [glutamine-hydrolyzing]	Synthesizes de novo asparagine from aspartate and glutamine	↑ mRNA expression in CRC cells (i.e. SW620 and HT-29) treated with anti-carcinogenic compounds.<br>↑ Expression in KRAS-mutated CRC cells/tissue, through the PI3K-AKT-mTOR pathway, is an important metabolic adaptation to support growth and survival. Silencing of ASNS diminished the proliferation of KRAS-mutant CRC cells *in vitro* and *in vivo*.[30-32]	1.732	0.004
SLC6A9	Sodium- and chloride-dependent glycine transporter 1	Terminates the action of glycine by its high affinity sodium-dependent reuptake into presynaptic terminals.	↑ mRNA expression when CRC cells (i.e. HCT-116 or RKO) were treated with protopanaxadiol, a cytotoxic metabolite from *P. quinquefolius* L., or Zinc during hypoxia.[33,34]	1.452	0.044
LRRN4	Leucine-rich repeat neuronal protein 4	May play an important role in hippocampus-dependent long-lasting memory.	Identified as a low abundance protein in CRC tissues.[35]	1.395	0.032
SERPINA3	Alpha-1-antichymotrypsin (SerpinA3)	Serine protease inhibitor, specific against neutrophil cathepsin G, mast cell chymase, and pancreatic chymotrypsin.	↓Expression in CRC samples (affected tissue, SW620 metastatic cells, HT-29 malignant colonospheres) when compared to paired normal samples.<br>↑ Expression in disseminated than localized CRC.<br>↑Levels in serum/plasma levels of CRC patients when compared to patients with polyps or healthy.[36-38]	1.376	0.012
TNC	Tenascin	Extracellular matrix protein that, in tumors, stimulates angiogenesis	↑ Expression in CRC tissue (mainly by cancer associated fibroblasts) that promote survival, proliferation, and invasiveness of CRC cells by induction of angiogenesis and metastasis, particularly to the liver.[39,40]	1.277	0.004

Supplementary Data

Supplementary Figures

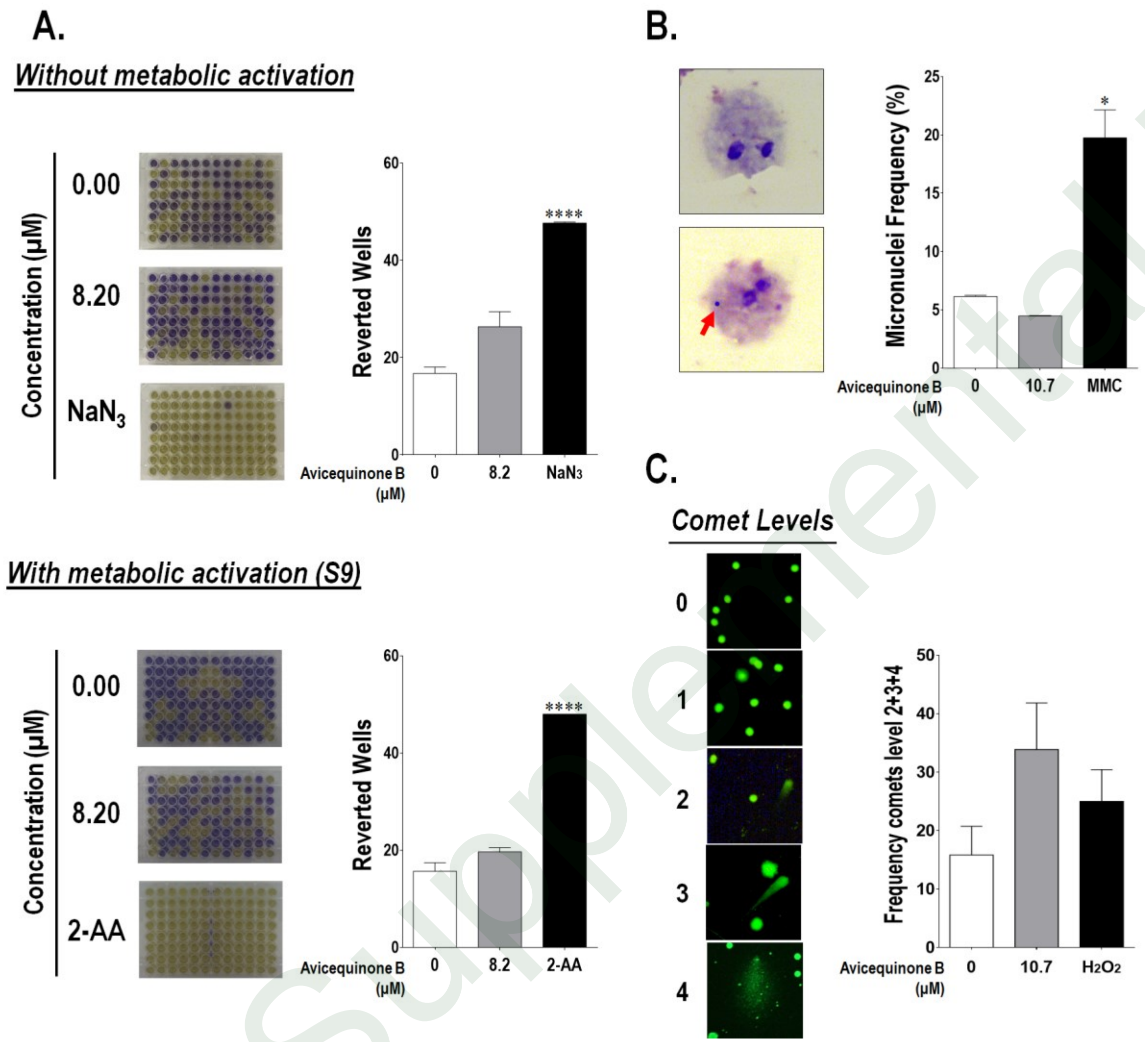

Figure S1. Avicequinone B did not induce mutagenic or genotoxic effects *in vitro*. (A) Ames test was performed using *Salmonella typhimurium* (TA100) treated with vehicle (DMSO, control), avicequinone B (8.20 µM), sodium azide (NaN3), or 2-aminoanthracene (2-AA), with and without S9 metabolic activation. Mutagenic effect was scored visually; yellow wells were counted as positive whereas purple wells counted as negative. The frequency of reverted wells is plotted in a bar graph. Results are representative of two independent experiments, and are expressed as the mean ± S.E.M. (*n*=4-11 per group). (****) *p*<0.0001 vs. control group. **(B)** Micronucleus assay was performed using 3T3-L1 fibroblasts treated with vehicle (DMSO, control), avicequinone B (10.7 µM) or mitomycin (MMC, 0.5 µM) for 30 h, before blocking the cytokinesis with cytochalasin. Representative pictures of a fibroblast Vs. micronucleated fibroblasts are shown; (Wright's Stain; 100X). The frequency of micronucleated fibroblasts is plotted in a bar graph. Results are representative of two independent experiments, and are expressed as the mean ± S.E.M. (*n*=5-10 per group). (*) *p*<0.05 vs. control group. **(C)** Comet assay was performed using 3T3-L1 fibroblasts treated with vehicle (DMSO, control), avicequinone B (10.7 µM) or hydrogen peroxide (150 µM) before neutral DNA-electrophoresis. Representative pictures of comet tails for each scored population assigned by three blinded investigators are shown (SYBER green stain; 10X). The frequency of comets with medium to complete DNA-damage was plotted in a bar graph. Results are representative of two independent experiments, and are expressed as the mean ± S.E.M. (*n*=4 per group).

Supplemental Data

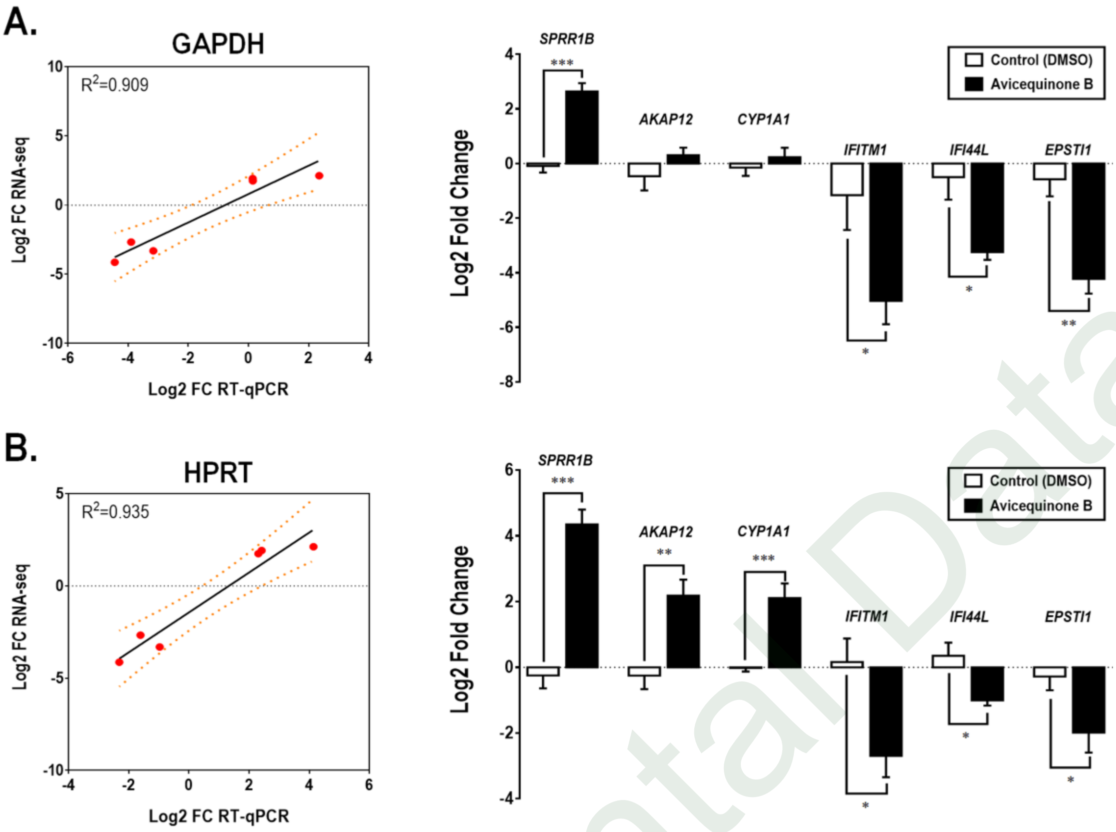

Figure S2. RT-qPCR validation of RNA-seq results. Six genes were selected to confirm the differential expression using the same samples used for RNA-seq (n=3) with RT-qPCR. Results were plotted as the correlation and linear regression analysis between RNA-seq and RT-qPCR data (Log2FC-fold change), in addition to the detailed results from the RT-qPCR analysis of selected genes when using **(A)** GAPDH or **(B)** HPRT as endogenous housekeeping genes. Data are expressed as mean ± S.E.M (n=3-6 per group), *$p<0.05$, **$p<0.01$, ***$p<0.001$, ****$p<0.0001$, as calculated by unpaired t-test with Welch´s correction when required.

Supplemental Data

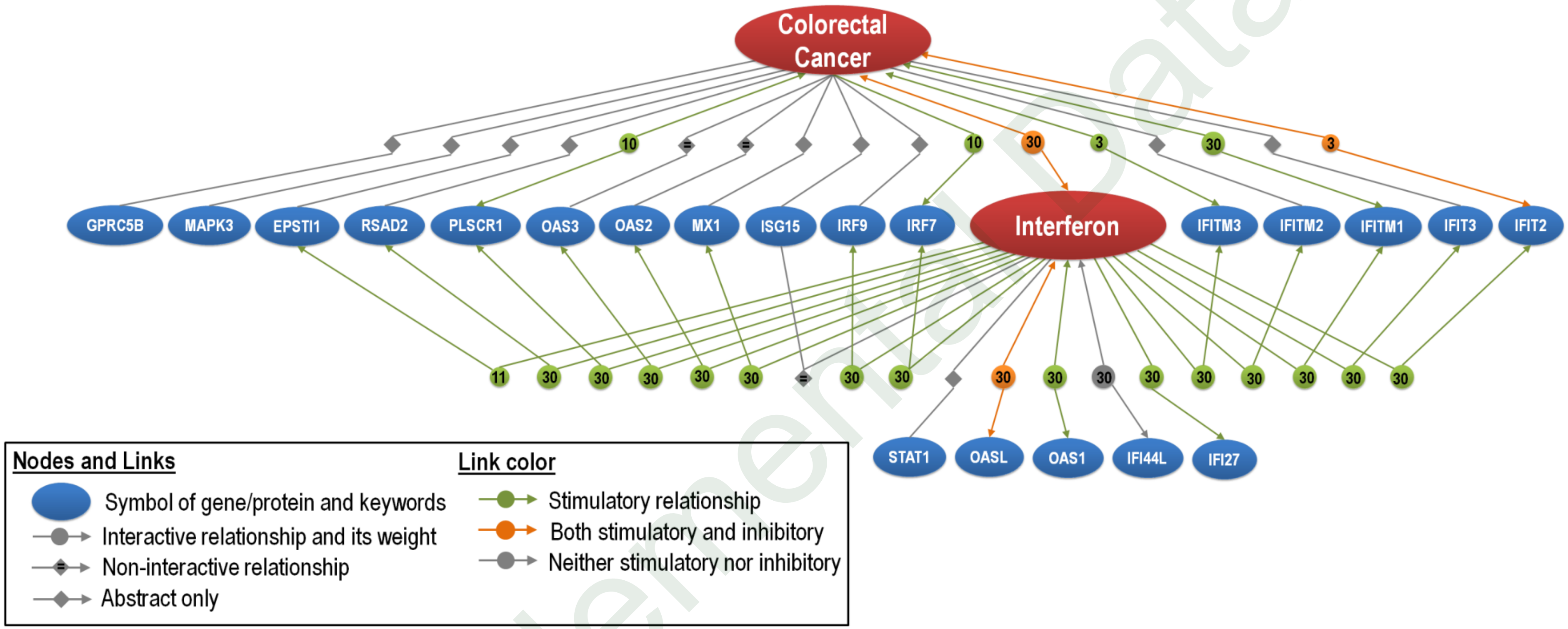

Figure S3. Colorectal cancer, interferon, and interferon regulated genes (IRGs) are strongly related. Text-mining was applied using Chillibot (http://www.chilibot.net/) searching for interactions between differentially expressed IRGs (CMPK2, EPSTI1, GPRC5B, IFI27, IFI44L, IFIT1, IFIT2, IFIT3, IFITM1, IFITM2, IFITM3, IRF7, IRF9, ISG15, MAPK3, MAPK11, MAPK12, MX1, OAS1, OAS2, OAS3, OASL, PLSCR1, RSAD2, STAT1, TNNC1), and the root keywords “Colorectal cancer” and “Interferon”. Updated December 07, 2018.

Supplemental Data

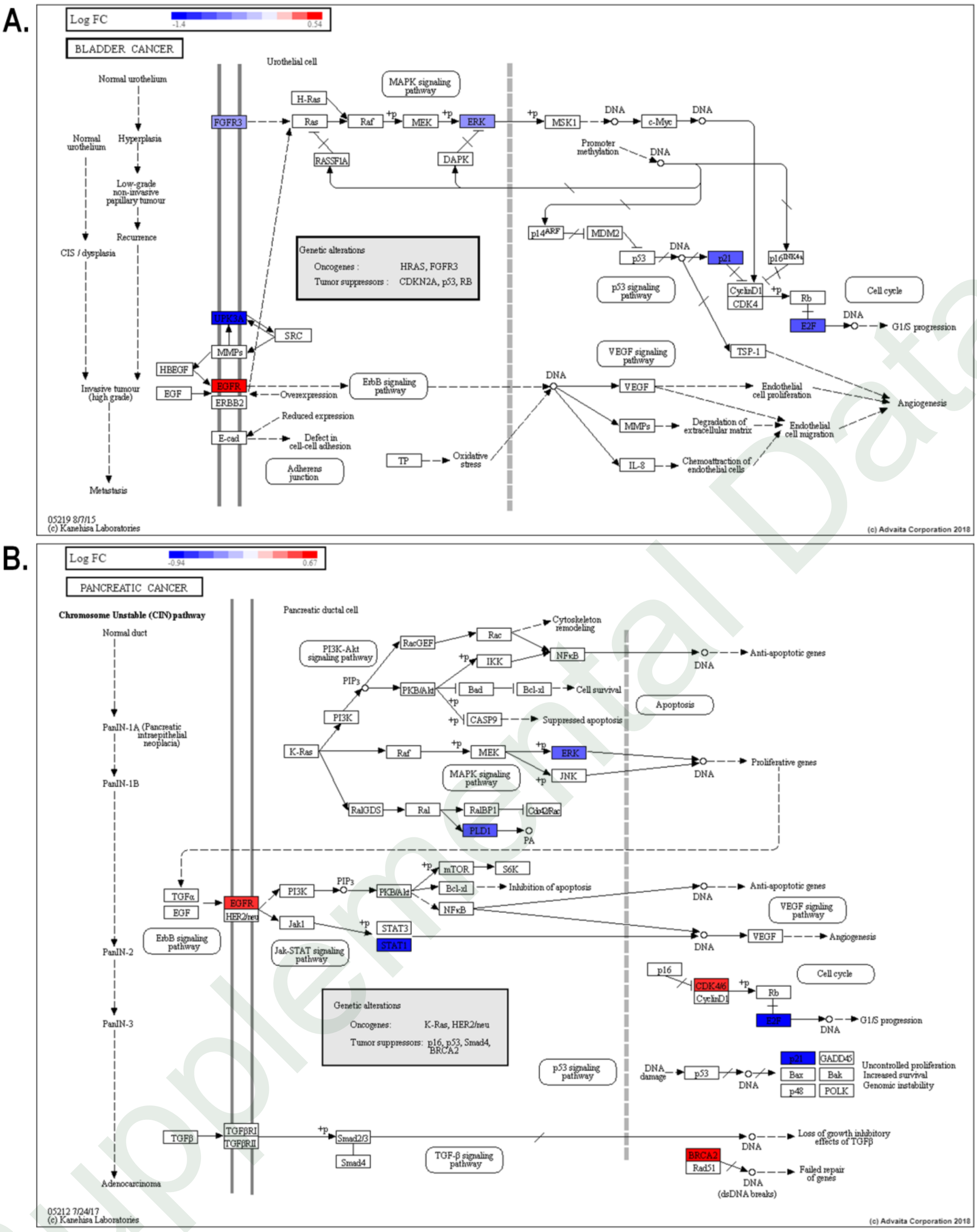

Supplemental Data

Figure S4. Avicequinone B significantly impacts the expression of targets within cancer signaling pathways. Differentially expressed genes (DEGs) between HT-29 cells treated with avicequinone B (8.20 µM) and vehicle (control) were analyzed using iPathwayGuide online software to identify that **(A)** Bladder Cancer, **(B)** Pancreatic Cancer, and **(C)** Breast Cancer were significantly impacted. The heat map at the top left shows the Log2 fold change (LogFC), where blue indicates down-regulated genes and red indicates up-regulated genes. Only those genes that meet our criteria of differential expression are shown (Log2 |(fold change)| ≥0.5, *p*-value ≤ 0.001, and *q*-value ≤ 0.05).

C.

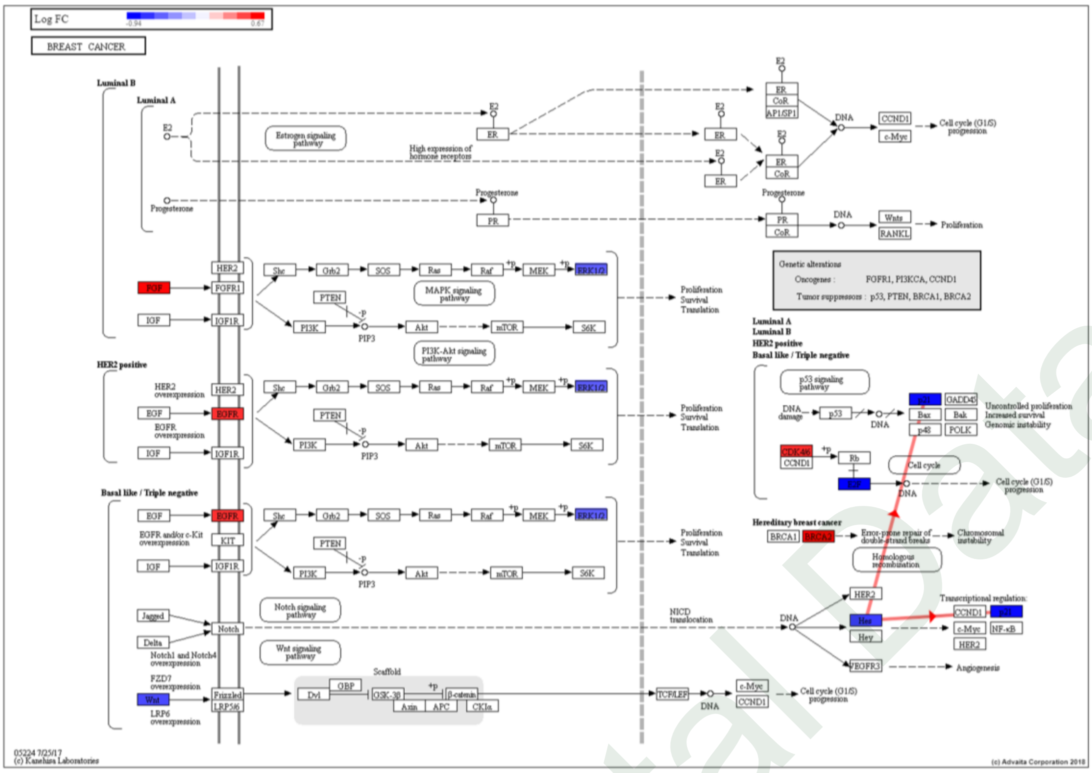

Figure S4. (Continuation)

Supplemental Data

A. B.

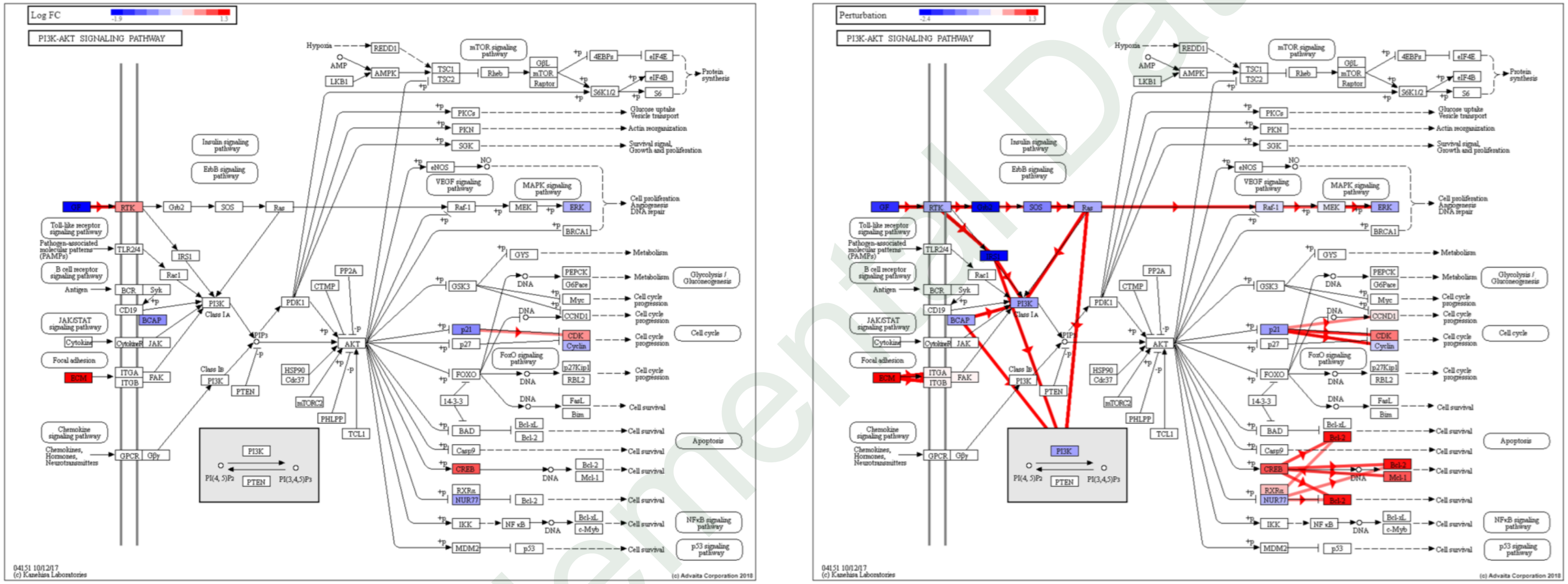

Figure S5. Avicequinone B significantly impacts the expression of targets within PI3K-AKT signaling pathway. Differentially expressed genes (DEGs) between HT-29 cells treated with compound avicequinone B (8.20 µM) and vehicle (control) were analyzed using iPathwayGuide online software. **(A)** Impact Analysis. The heat map at the top left shows the Log2 fold change (LogFC), where blue indicates down-regulated genes and red indicates up-regulated genes. Only those genes that meet our criteria of differential expression are shown (Log2 |(fold change)| ≥0.5, *p*-value ≤ 0.001, and *q*-value ≤ 0.05). **(B)** Perturbation analysis. The heat map at the top left shows the predicted perturbation (FC + accumulation), where blue indicates down-regulated genes and red indicates up-regulated genes, bold red highlighted arrows indicates coherent cascades.

Supplemental Data

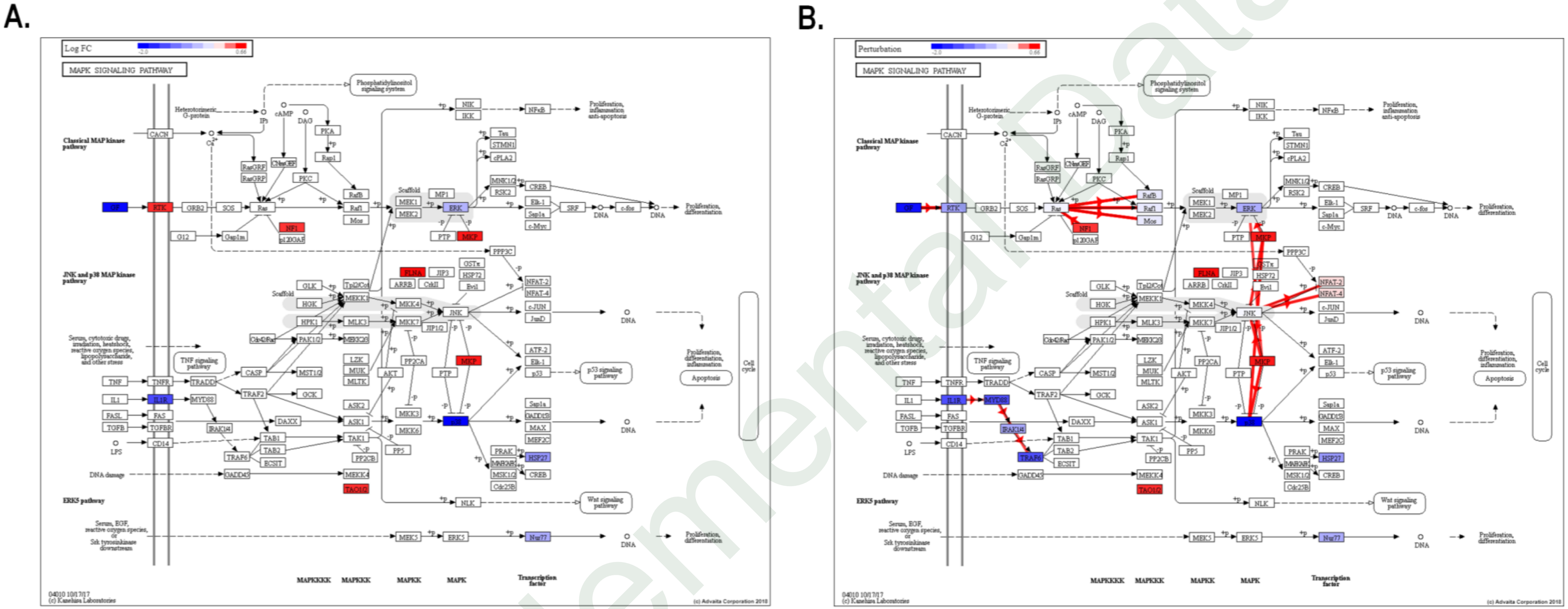

Figure S6. Avicequinone B significantly impacts the expression of targets within MAPK signaling pathway. Differentially expressed genes (DEGs) between HT-29 cells treated with compound avicequinone B (8.20 µM) and vehicle (control) were analyzed using iPathwayGuide online software. **(A)** Impact Analysis. The heat map at the top left shows the Log2 fold change (LogFC), where blue indicates down-regulated genes and red indicates up-regulated genes. Only those genes that meet our criteria of differential expression are shown (Log2 |(fold change)| ≥0.5, *p*-value ≤ 0.001, and *q*-value ≤ 0.05). **(B)** Perturbation analysis. The heat map at the top left shows the predicted perturbation (FC + accumulation), where blue indicates down-regulated genes and red indicates up-regulated genes, bold red highlighted arrows indicate coherent cascades.

Supplemental Data

1. References

1. Bolger AM, Lohse M, Usadel B. Trimmomatic: A flexible trimmer for illumina sequence data. *Bioinformatics* 2014;30(15):2114-20. doi: 10.1093/bioinformatics/btu170
2. Trapnell C, Pachter L, Salzberg SL. Tophat: Discovering splice junctions with rna-seq. *Bioinformatics* 2009;25(9):1105-11. doi: 10.1093/bioinformatics/btp120
3. Trapnell C, Roberts A, Goff L, Pertea G, Kim D, Kelley DR, et al. Differential gene and transcript expression analysis of rna-seq experiments with tophat and cufflinks. *Nature protocols* 2012;7(3):562-78. doi: 10.1038/nprot.2012.016
4. Szklarczyk D, Franceschini A, Wyder S, Forslund K, Heller D, Huerta-Cepas J, et al. String v10: Protein-protein interaction networks, integrated over the tree of life. *Nucleic acids research* 2015;43(Database issue):D447-52. doi: 10.1093/nar/gku1003
5. Shannon P, Markiel A, Ozier O, Baliga NS, Wang JT, Ramage D, et al. Cytoscape: A software environment for integrated models of biomolecular interaction networks. *Genome research* 2003;13(11):2498-504. doi: 10.1101/gr.1239303
6. Andreu P, Colnot S, Godard C, Laurent-Puig P, Lamarque D, Kahn A, et al. Identification of the *ifitm* family as a new molecular marker in human colorectal tumors. *Cancer Research* 2006;66(4):1949. doi: 10.1158/0008-5472.CAN-05-2731
7. He JD, Luo HL, Li J, Feng WT, Chen LB. Influences of the interferon induced transmembrane protein 1 on the proliferation, invasion, and metastasis of the colorectal cancer sw480 cell lines. *Chinese medical journal* 2012;125(3):517-22.
8. He J, Li J, Feng W, Chen L, Yang K. Prognostic significance of inf-induced transmembrane protein 1 in colorectal cancer. *International journal of clinical and experimental pathology* 2015;8(12):16007-13.
9. Yu F, Xie D, Ng SS, Lum CT, Cai M-Y, Cheung WK, et al. Ifitm1 promotes the metastasis of human colorectal cancer via cav-1. *Cancer Letters* 2015;368(1):135-43. doi: https://doi.org/10.1016/j.canlet.2015.07.034
10. Li X, Gill R, Cooper NG, Yoo JK, Datta S. Modeling microrna-mrna interactions using pls regression in human colon cancer. *BMC Medical Genomics* 2011;4(1):44. doi: 10.1186/1755-8794-4-44
11. Virag P, Fischer-Fodor E, Perde-Schrepler M, Brie I, Tatomir C, Balacescu L, et al. Oxaliplatin induces different cellular and molecular chemoresistance patterns in colorectal cancer cell lines of identical origins. *BMC Genomics* 2013;14(1):480. doi: 10.1186/1471-2164-14-480
12. Wagner S, Vlachogiannis G, De Haven Brandon A, Valenti M, Box G, Jenkins L, et al. Suppression of interferon gene expression overcomes resistance to mek inhibition in kras-mutant colorectal cancer. *Oncogene* 2018. doi: 10.1038/s41388-018-0554-z
13. Tang H, Guo Q, Zhang C, Zhu J, Yang H, Zou YL, et al. Identification of an intermediate signature that marks the initial phases of the colorectal adenoma-carcinoma transition. *International journal of molecular medicine* 2010;26(5):631-41. doi: https://doi.org/10.3892/ijmm_00000508
14. Kim S-C, Hong C-W, Jang S-G, Kim Y-A, Yoo B-C, Shin Y-K, et al. Establishment and characterization of paired primary and peritoneal seeding human colorectal cancer cell lines: Identification of genes that mediate metastatic potential. *Translational Oncology* 2018;11(5):1232-43. doi: 10.1016/j.tranon.2018.07.014
15. Yu W, Lin Z, Pastor DM, Hegarty JP, Chen X, Kelly AA, et al. Genes regulated by nkx2-3 in sporadic and inflammatory bowel disease-associated colorectal cancer cell lines. *Digestive diseases and sciences* 2010;55(11):3171-80. doi: 10.1007/s10620-010-1138-0

Supplemental Data

16. Heckmann D, Maier P, Laufs S, Li L, Sleeman JP, Trunk MJ, et al. The disparate twins: A comparative study of cxcr4 and cxcr7 in sdf-1α–induced gene expression, invasion and chemosensitivity of colon cancer. *Clinical Cancer Research* 2014;20(3):604-16. doi: 10.1158/1078-0432.ccr-13-0582
17. Slattery ML, Pellatt AJ, Lee FY, Herrick JS, Samowitz WS, Stevens JR, et al. Infrequently expressed mirnas influence survival after diagnosis with colorectal cancer. *Oncotarget* 2017;8(48):83845-59. doi: 10.18632/oncotarget.19863
18. Dai W, Li Y, Mo S, Feng Y, Zhang L, Xu Y, et al. A robust gene signature for the prediction of early relapse in stage i-iii colon cancer. *Molecular oncology* 2018;12(4):463-75. doi: 10.1002/1878-0261.12175
19. Giarnieri E, Alderisio M, Valli C, Vecchione A, Forte A, Turano R, et al. Overexpression of ndp kinase nm23 associated with ploidy image analysis in colorectal cancer. *Anticancer research* 1995;15(5B):2049-53.
20. Yamashita K, Katoh H, Watanabe M. The homeobox only protein homeobox (hopx) and colorectal cancer. *International journal of molecular sciences* 2013;14(12):23231-43. doi: 10.3390/ijms141223231
21. Oltedal S, Skaland I, Maple-Grødem J, Tjensvoll K, Janssen EAM, Gilje B, et al. Expression profiling and intracellular localization studies of the novel proline-, histidine-, and glycine-rich protein 1 suggest an essential role in gastro-intestinal epithelium and a potential clinical application in colorectal cancer diagnostics. *BMC Gastroenterology* 2018;18(1):26. doi: 10.1186/s12876-018-0752-8
22. He P, Li K, Li SB, Hu TT, Guan M, Sun FY, et al. Upregulation of akap12 with hdac3 depletion suppresses the progression and migration of colorectal cancer. *International journal of oncology* 2018;52(4):1305-16. doi: 10.3892/ijo.2018.4284
23. Liu W, Guan M, Su B, Ye C, Li J, Zhang X, et al. Quantitative assessment of akap12 promoter methylation in colorectal cancer using methylation-sensitive high resolution melting: Correlation with duke's stage. *Cancer biology & therapy* 2010;9(11):862-71. doi: 10.4161/cbt.9.11.11633
24. Liu W, Guan M, Hu T, Gu X, Lu Y. Re-expression of akap12 inhibits progression and metastasis potential of colorectal carcinoma *in vivo* and *in vitro*. *PloS one* 2011;6(8):e24015. doi: 10.1371/journal.pone.0024015
25. Luque-Garcia JL, Martinez-Torrecuadrada JL, Epifano C, Canamero M, Babel I, Casal JI. Differential protein expression on the cell surface of colorectal cancer cells associated to tumor metastasis. *Proteomics* 2010;10(5):940-52. doi: 10.1002/pmic.200900441
26. Willis AJ, Indra R, Wohak LE, Sozeri O, Feser K, Mrizova I, et al. The impact of chemotherapeutic drugs on the cyp1a1-catalysed metabolism of the environmental carcinogen benzo[a]pyrene: Effects in human colorectal hct116 tp53(+/+), tp53(+/−) and tp53(−/−) cells. *Toxicology* 2018;398-399:1-12. doi: https://doi.org/10.1016/j.tox.2018.02.006
27. Pande M, Amos CI, Osterwisch DR, Chen J, Lynch PM, Broaddus R, et al. Genetic variation in genes for the xenobiotic-metabolizing enzymes *cyp1a1*, *ephx1*, *gstm1*, *gstt1*, and *gstp1* and susceptibility to colorectal cancer in lynch syndrome. *Cancer Epidemiology Biomarkers & Prevention* 2008;17(9):2393-401. doi: 10.1158/1055-9965.epi-08-0326
28. Zheng Y, Wang J-J, Sun L, Li H-L. Association between cyp1a1 polymorphism and colorectal cancer risk: A meta-analysis. *Molecular Biology Reports* 2012;39(4):3533-40. doi: 10.1007/s11033-011-1126-2

Supplemental Data

29. Chou CK, Fan CC, Lin PS, Liao PY, Tung JC, Hsieh CH, et al. Sciellin mediates mesenchymal-to-epithelial transition in colorectal cancer hepatic metastasis. *Oncotarget* 2016;7(18):25742-54. doi: 10.18632/oncotarget.8264
30. Doleman JF, Eady JJ, Elliott RM, Foxall RJ, Seers J, Johnson IT, et al. Identification of the eph receptor pathway as a novel target for eicosapentaenoic acid (epa) modification of gene expression in human colon adenocarcinoma cells (ht-29). *Nutrition & Metabolism* 2010;7(1):56. doi: 10.1186/1743-7075-7-56
31. Lundemo AG, Pettersen CH, Berge K, Berge RK, Schønberg SA. Tetradecylthioacetic acid inhibits proliferation of human sw620 colon cancer cells - gene expression profiling implies endoplasmic reticulum stress. *Lipids in Health and Disease* 2011;10(1):190. doi: 10.1186/1476-511x-10-190
32. Toda K, Kawada K, Iwamoto M, Inamoto S, Sasazuki T, Shirasawa S, et al. Metabolic alterations caused by kras mutations in colorectal cancer contribute to cell adaptation to glutamine depletion by upregulation of asparagine synthetase. *Neoplasia* 2016;18(11):654-65. doi: 10.1016/j.neo.2016.09.004
33. Zhang Z, Li Z, Wu X, Zhang C-F, Calway T, He T-C, et al. Trail pathway is associated with inhibition of colon cancer by protopanaxadiol. *Journal of Pharmacological Sciences* 2015;127(1):83-91. doi: https://doi.org/10.1016/j.jphs.2014.11.003
34. Sheffer M, Simon AJ, Jacob-Hirsch J, Rechavi G, Domany E, Givol D, et al. Genome-wide analysis discloses reversal of the hypoxia-induced changes of gene expression in colon cancer cells by zinc supplementation. *Oncotarget* 2011;2(12):1191-202. doi: 10.18632/oncotarget.395
35. Lim SR, Gooi BH, Gam LH. Identification of low abundance proteins in colorectal cancer tissues. *Cancer biomarkers : section A of Disease markers* 2012;12(4):185-98. doi: 10.3233/CBM-130307
36. Dimberg J, Strom K, Lofgren S, Zar N, Hugander A, Matussek A. Expression of the serine protease inhibitor serpina3 in human colorectal adenocarcinomas. *Oncology letters* 2011;2(3):413-8. doi: 10.3892/ol.2011.280
37. Long NP, Lee WJ, Huy NT, Lee SJ, Park JH, Kwon SW. Novel biomarker candidates for colorectal cancer metastasis: A meta-analysis of *in vitro* studies. *Cancer informatics* 2016;15(Suppl 4):11-7. doi: 10.4137/CIN.S40301
38. Peltier J, Roperch J-P, Audebert S, Borg J-P, Camoin L. Quantitative proteomic analysis exploring progression of colorectal cancer: Modulation of the serpin family. *Journal of Proteomics* 2016;148:139-48. doi: https://doi.org/10.1016/j.jprot.2016.07.031
39. Murakami T, Kikuchi H, Ishimatsu H, Iino I, Hirotsu A, Matsumoto T, et al. Tenascin c in colorectal cancer stroma is a predictive marker for liver metastasis and is a potent target of mir-198 as identified by microrna analysis. *British journal of cancer* 2017;117(9):1360-70. doi: 10.1038/bjc.2017.291
40. Li M, Peng F, Li G, Fu Y, Huang Y, Chen Z, et al. Proteomic analysis of stromal proteins in different stages of colorectal cancer establishes tenascin-c as a stromal biomarker for colorectal cancer metastasis. *Oncotarget* 2016;7(24):37226-37. doi: 10.18632/oncotarget.9362

Supplemental Data
